# Activation of Toll‐like receptor 7 provides cardioprotection in septic cardiomyopathy‐induced systolic dysfunction

**DOI:** 10.1002/ctm2.266

**Published:** 2021-01-01

**Authors:** Xie Saiyang, Wu Qingqing, Xu man, Liu Chen, Zhang Min, Xing Yun, Shi Wenke, Wu Haiming, Zeng Xiaofeng, Chen Si, Guo Haipeng, Deng Wei, Tang Qizhu

**Affiliations:** ^1^ Department of Cardiology Renmin Hospital of Wuhan University Wuhan People's Republic of China; ^2^ Hubei Key Laboratory of Metabolic and Chronic Diseases Wuhan People's Republic of China; ^3^ Department of Cardiology The Fifth Affiliated Hospital of Xinjiang Medical University Ürümqi China; ^4^ Key Laboratory of Cardiovascular Remodeling and Function Research, Chinese Ministry of Education and Chinese Ministry of Health Qilu Hospital of Shandong University Jinan China; ^5^ Department of Critical Care Medicine Qilu Hospital of Shandong University Jinan People's Republic of China

**Keywords:** Ca^2+^‐handling, cardiac dysfunction, septic cardiomyopathy, TLR7

## Abstract

**Background:**

As a pattern recognition receptor, Toll‐like receptor 7 (TLR7) widely presented in the endosomal membrane of various cells. However, the precise role and mechanism of TLR7 in septic cardiomyopathy remain unknown. This study aims to determine the role of TLR7 in cardiac dysfunction during sepsis and explore the mechanism of TLR7 in septic cardiomyopathy.

**Methods:**

We generated a mouse model of septic cardiomyopathy by challenging with lipopolysaccharide (LPS). TLR7‐knockout (TLR7^−/−^), wild‐type (WT) mice, cardiac‐specific TLR7‐transgenic (cTG‐TLR7) overexpression, and littermates WT (LWT) mice were subjected to septic model. Additionally, to verify the role and mechanism of TLR7 in vitro, we transfected neonatal rat ventricular myocytes (NRVMs) with Ad‐TLR7 and TLR7 siRNA before LPS administration. The effects of TLR7 were assessed by Ca^2+^ imaging, western blotting, immunostaining, and quantitative real‐time polymerase chain reaction (qPCR).

**Results:**

We found that TLR7 knockout markedly exacerbated sepsis‐induced systolic dysfunction. Moreover, cardiomyocytes isolated from TLR7^−/−^ mice displayed weaker Ca^2+^ handling than that in WT mice in response to LPS. Conversely, TLR7 overexpression alleviated LPS‐induced systolic dysfunction, and loxoribine (TLR7‐specific agonist) improved LPS‐induced cardiac dysfunction. Mechanistically, these optimized effects were associated with enhanced the adenosine (cAMP)‐protein kinase A (PKA) pathway, which upregulated phosphorylate‐phospholamban (p‐PLN) (Ser16) and promoted sarco/endoplasmic reticulum Ca^2+^ ATPase (Serca) and Ryanodine Receptor 2 (RyR2) expression in the sarcoplasmic reticulum (SR), and ultimately restored Ca^2+^ handling in response to sepsis. While improved Ca^2+^ handling was abrogated after H89 (a specific PKA inhibitor) pretreatment in cardiomyocytes isolated from cTG‐TLR7 mice. Consistently, TLR7 overexpression improved LPS‐induced Ca^2+^‐handling decrement in NRVMs. Nevertheless, TLR7 knockdown showed a deteriorative phenotype.

**Conclusions:**

Our data demonstrated that activation of TLR7 protected against sepsis‐induced cardiac dysfunction through promoting cAMP‐PKA‐PLN pathway, and we revealed that TLR7 might be a novel therapeutic target to block the septic cardiomyopathy and support systolic function during sepsis.

## Graphical abstract

Our study explored the effect of TLR7 activation, by which TLR7 modulated Ca2+ handling in septic cardiomyopathy. TLR7 protected against sepsis‐induced cardiac dysfunction through activation of cAMP‐PKA‐PLN pathway. Targeting this newly identified TLR7 may yield novel interventional solutions against septic cardiomyopathy.

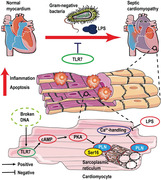

**Abbreviations**
AMCMs: adult mice cardiomyocytes
ATP2A: Ca2+‐ transporting ATPase 2A
CaMKII: Ca2+/calmodulin‐dependent protein kinases
cAMP: adenosine
EF: ejection fraction
GLI: glioma‐associated oncogene
HMGB1: high mobility group box‐1 protein
LPS: lipopolysaccharide
LWT: littermates wild‐type
NRVM: neonatal rat ventricular myocyte
NS: normal saline
OCT: optimal cutting temperature
PKA: protein kinase A
PKC: protein kinase C
PLN: phospholamban
RyR2: ryanodine receptor 2
Serca: sarcoplasmic reticulum Ca2+‐ATPase
SR: sarcoplasmic reticulum
SR/ER: sarcoplasmic/endoplasmic reticulum
TLR7: Toll‐like receptor 7

## 1 INTRODUCTION

Sepsis is characterized by multiple organ failure caused by toxins produced by pathogenic microorganisms.^1^ Septic cardiomyopathy accounts for 18‐65% of all septic complications, and high mortality rates of around 36‐55% globally.^1^, ^2^ Some of the factors that contribute to septic cardiomyopathy include apoptosis, mitochondrial dysfunction, abnormal regulation of intracellular Ca^2+^ transporter, endotoxins, inflammatory factors, immune responses, and energy metabolism disorders.^3^, ^4^, ^5^, ^6^ However, the mechanisms of septic cardiomyopathy remain unclear.

Several studies report that abnormal host response mediated by Toll‐like receptor (TLR) contributes to the pathogenesis of sepsis.^7^, ^8^ Recently, TLR4 and TLR2 have been shown to play a critical role in sepsis‐induced cardiac dysfunction.^7^, ^9^, ^10^ Moreover, TLR7, a pattern recognition receptor is found in the endosomal membrane.^11^ Studies have shown that TLR7 is present in the endoplasmic reticulum (ER) in unstimulated cells, where it is transmitted into the endosomes and bound to the ligand recognition in case that bacterial toxins or single‐stranded RNA fragments invaded the cell.^12^, ^13^, ^14^, ^15^ TLR7 has recently been suggested to be partly responsible for cardiac dysfunction.^16^, ^17^ However, the role and mechanism of TLR7 in septic cardiomyopathy remain unknown.

Sarcoplasmic/ER (SR/ER)Ca2+ ATPase (Serca) is an important ATPase that maintains Ca^2+^ homeostasis, by regulating Ca^2+^ uptake from the cytosol into the sarcoplasmic reticulum (SR).^18^ This complex process is regulated by Ryanodine Receptor 2 (RyR2) channels, and it is essential for the excitation‐contraction coupling to balance Ca2+ homeostasis, while abnormal Ca^2+^ handling caused by SR dysfunction in cardiomyocytes results in systolic dysfunction.^19^ Moreover, previous studies have suggested that phospholamban (PLN) inhibits Ca^2+^ uptake from the cytosol into the sarcoplasmic reticulum by suppressing Serca activity and results in decreased Ca^2+^ transient in cardio‐myocytes, and phosphorylated PLN restores Serca activity.^20^ Other studies have shown that the cAMP‐protein kinase A (PKA) pathway is significant in Ca^2+^ handling by regulating phosphorylation and dephosphorylation of PLN.^21^, ^22^ Furthermore, the relationship between TLR7 and Ca^2+^ homeostasis has become a recent research hotspot. Kim et al^23^ found that imiquimod (IQ), an agonist of TLR7, increased intracellular Ca2+ flux in the dorsal root ganglion neurons. Moreover, Gamo et al^24^ suggested that both TLR7 and Serca2 were located in the tracheal submucosal gland cells and resulted in rapid attenuation of acetylcholine‐induced, Ca2+ dependent ionic currents by promoting Serca2‐mediated Ca2+ clearance. Additionally, De Marcken et al^25^ reported that TLR7, but not TLR8, activation stimulated Ca2+ flux in monocytes that prevented type I interferon (IFN‐1) responses when human monocytes were incubated with the virus. However, the correlation between TLR7 and Ca^2+^ handling in septic cardiomyopathy has been largely ignored.

Herein, our data showed that TLR7 expression was upregulated in cardiomyocytes in response to sepsis, and TLR7 activation further activates the cAMP‐PKA pathway in the sarcoplasmic reticulum. Subsequently, PKA directly phosphorylates Serine 16 of the PLN instead of Threonine 17, which results in enhanced Serca and RyR2 expression in the sarcoplasmic reticulum, and simultaneously facilitates excitation‐contraction coupling in cardiomyocytes. On the other hand, TLR7 deficiency exacerbates systolic dysfunction in response to sepsis. Therefore, in this study, we hypothesized that TLR7 may be a potential therapeutic target for septic cardiomyopathy by improving Ca^2+^ handling in cardiomyocytes.

## 2 METHODS

All animal experiments were approved by the Committee of Renmin Hospital of Wuhan University on the ethic of animal experiments and complied with the Guide for the Care and Use of Laboratory Animals (National Institutes of Health publication number: 85‐23, revised 1996).

### 2.1 Animals and treatment

A total of 8‐10 weeks old C57/B6J mice (male; 23.5‐27.5 g) were purchased from the Institute of Laboratory Animal Science, Chinese Academy of Medical Sciences (Beijing, China), kept in a quarantine room for a week to adapt to the environment. All mice were housed under specific environmentally controlled (temperature: 20‐25 °C; Humidity: 50 ± 5%) barrier conditions and fed on rodent diet ad libitum. Furthermore, cardiomyocyte‐specific TLR7 overexpression (TLR7‐cTG, bought from the Jackson Laboratory) and TLR7 knockout (TLR7^−/−^, C57/B6J background, bought from the Jackson Laboratory) mice were utilized in this study as well. The detail of genetic background and generation of TLR7^−/−^ and TLR7‐cTG mice is provided in the online supplementary material.

Mice aged 10 weeks (23.5‐27.5 g) were randomly assigned into four groups: NS‐WT group (n = 12), NS‐TLR7^−/−^ group (n = 12), lipopolysaccharide (LPS)‐WT group (n = 20), and LPS‐TLR7^−/−^ group (n = 20). Mice in each group underwent an intraperitoneal injection of LPS (Sigma,10 mg/kg) or saline equally. Furthermore, 10 weeks old TLR7‐cTG and LWT mice (23.5‐27.5g) were randomly divided into four groups: NS‐LWT group (n = 12), NS‐cTG‐TLR7 group (n = 12), LPS‐LWT group (n = 20), and LPS‐cTG‐TLR7 group (n = 20). Mice in each group were injected intraperitoneally with LPS (10 mg/kg) or saline. Additional details are provided in the supplementary material online.

### 2.2 Analysis of cardiac function

Echocardiography was performed following LPS stimulation for 6 hours, 12 hours, and 24 hours. In our previous study,^26^ mice were anesthetized (2% isoflurane) and analyzed with a 10‐MHz linear array ultrasound transducer equipped with a 30‐MHz probe (MS550D) (Visual Sonics, Toronto, Canada). Echocardiographic parameters were measured under the long‐axis M‐mode when the heart rate was about 450 bpm, including LV internal diameter at end‐systole (LVIDs), LV end‐diastolic internal diameter (LVIDd), and interventricular septum thickness (IVSs). The fractional shortening (FS) was evaluated using the formula: LVFS = (LVIDd‐LVIDs) × 100/LVIDd. The LV ejection fraction (LVEF) was assessed using the Teichholtz formula: LVEF = ([100‐Y] × 0.15) +Y; Y = (LVIDd^2^‐LVIDs^2^) × 100/LVIDd^2^.

Pressure‐volume (PV) loop parameters were recorded using the Power Lab PV system as previously described.^26^ Briefly, hemodynamic parameters were monitored by Power Lab system (AD Instruments Ltd., Oxford, UK) equipped with a 1.4‐French Millar PV catheter (SPR‐839; Millar Instruments, Houston, TX). Hemodynamic parameters were detected and included the PV curve, end‐systole volume (ESV), and end‐diastolic volume (EDV).

### 2.3 Histology

After LPS stimulation for 24 hours, mice were euthanized, and then the heart was harvested for histological analysis with paraffin imbedded sections. The 5 µm thick sections were used for morphological analysis. Histologically, hematoxylin‐eosin (H&E) staining was performed to evaluate the cardiomyocytes arrangement. For immunohistochemistry, the paraffin sections were heated in a pressure cooker for antigen retrieval and incubated with CD68 antibody (ab125212, Abcam) or TUNEL probe. Goat anti‐rabbit EnVisionTM**+**/horseradish peroxidase reagent was added, followed by subsequent staining with DAB detection kit (Gene Tech, Shanghai, China). For immunofluorescence assay, the sections were incubated with primary antibodies diluted in phosphate buffered saline (PBS), supplemented with 1% BSA and 1% Triton X‐100 overnight at 4°C. The following primary antibodies were used: Serca (Abcam, ab2816) and RyR2 (Abcam, ab2868). The primary antibodies were detected with Alexa Fluor 488 goat anti‐mouse/rabbit IgG and Alexa Fluor 568 goat anti‐mouse/rabbit IgG and incubated at 37°C for 60 minutes. Quantification was performed with Image‐Pro Plus 6.0 software (Media Cybernetics, Bethesda, MD).

### 2.4 Isolation of adult mice cardiomyocytes

Adult mice cardiomyocytes (AMCMs) were harvested as previously described.^27^ Briefly, after LPS administration for 24 hours, the heart was removed by aseptic surgery, placed in a Ca^2+^‐free Tyrode's solution at 4°C, and the pre‐cooling heart was hanged in the Langendorff isolated cardiac perfusion system. The aorta was reversed and perfused with Tyrode's solution promoted by 95% O_2_ **+** 5% CO_2_ after aortic cannulation. Firstly, the excised heart was immersed and perfused with Ca^2+^‐free Tyrode's solution for 3 minutes, and enzyme digestive solution (type II collagenase, 1.5 g/L) for enzymatic digestion, which made the heart swell and softens. The ventricle was cut, digested, filtered, centrifuged, suspended, and separated into tissue pieces of 1∼2 mm^3^ in size to obtain cardio‐myocytes. Morphology of AMCMs was observed under an inverted microscope, and the length and width were evaluated using Image‐Pro Plus 6.0 (Media Cybernetics, Bethesda, MD). Furthermore, cell shortening was detected using a video microscopy motion detector system.

### 2.5 Isolation of neonatal rat cardiomyocytes

Neonatal rat ventricular myocytes (NRVMs) were obtained from the hearts of 1‐2 days old Sprague‐Dawley rats as previously described.^26^ Hearts of 1‐ to 3‐day‐old pups were excised and the atria removed. The ventricles were digested 3‐4 times at 37°C for 5 minutes with 0.125% trypsin freshly suspended in calcium‐free HBSS solution, pH 7.4. The supernatants containing isolated cells were collected from each digestion, and an equal volume of serum‐rich medium was added to stop the digestion. Isolated cells were collected and centrifuged at 800 rpm for 5 minutes to separate non‐myocardial cells. The resuspended cells were pre‐plated twice for 30 minutes to further reduce fibroblast contamination. NRVMs were finally plated in 3 cm dishes or 24‐well plates at a density of 0.3‐1 × 10^6^ cells/well, and 15% fetal bovine serum was added into the medium and cultured at 37°C and 5% CO_2_.

### 2.6 Ca^2+^ measurements

After isolation of neonatal and adult cardiomyocytes, they were loaded with a Ca^2+^ fluorophore (10 µmol/L Fluo‐4 AM, Invitrogen), observed with Leica AF6000 fluorescence microscope, and Ca^2+^ handling detected as previously described.^27^ Briefly, systolic Ca^2+^ transient was recorded in steady‐state conditions under constant field stimulation (1.0 Hz, 2.0‐ms duration, stimulation voltage set to 1.5 times the threshold). The Ca^2+^ content of the sarcoplasmic reticulum and the amplitude of caffeine‐induced Ca^2+^ transient were also recorded. The PKA inhibitor (H89, 10µM, MCE) was administered as previously described.^27^ Additional details are provided in the supplementary material online.

### 2.7 Adenovirus infection and siRNA transfection

NRVMs were infected with adenovirus encoding TLR7 (Vigene Biosciences, Jinan, China) particles at MOI of 50 for 12 hours, and Ad‐LacZ was used as a control. TLR7 mRNA was detected by quantitative real‐time polymerase chain reaction (qPCR). Small siRNA oligos (RiboBio, Guangzhou, China) were transfected with Lipo‐6000 reagent (Beyotime Biotechnology, China) following the manufacturer's instructions. Total RNA was extracted after 24 hours to determine the effectiveness of siRNA transfection by qPCR. Additional details are provided in the supplementary material online.

### 2.8 Statistical analysis

Prism software (GraphPad software Inc., 8.0 La Jolla, CA) was used to perform all the statistical analysis. *P* values were calculated with one‐way ANOVA or two‐way ANOVA followed by Bonferroni post‐hoc test or Student's *t*‐test as appropriate. The log‐rank test was used to determine survival analysis. Data were presented as Mean ± SEM; *P* < .05 was considered to be statistically significant.

## 3 RESULTS

### 3.1 TLR7 expression is upregulated in response to LPS in vivo and in vitro

**FIGURE 1:**
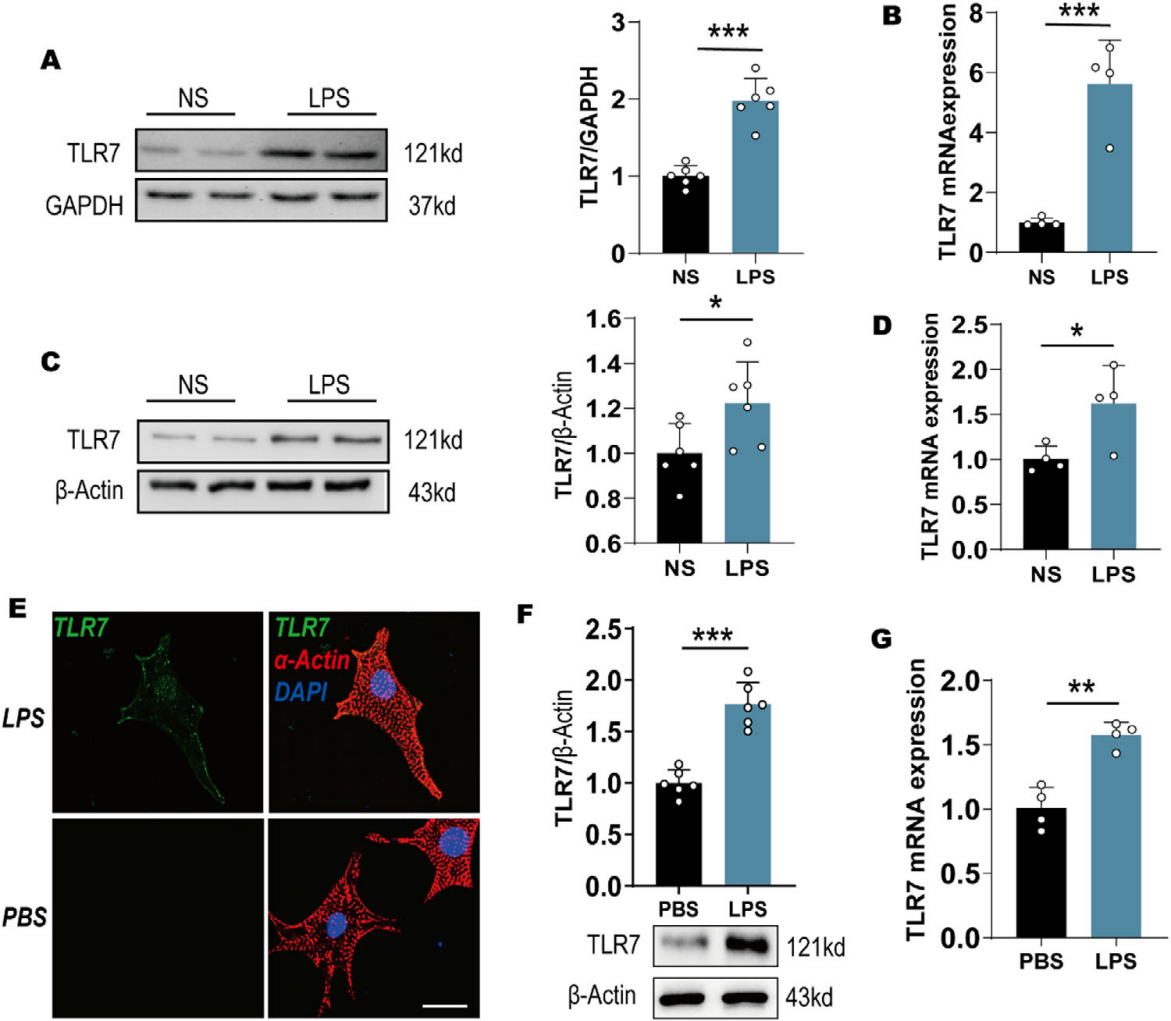
TLR7 expression was upregulated in response to LPS in vivo and in vitro. C57BL/6J mice (n = 24) were subjected to LPS (10 mg/kg) or saline for 24 hours. A, Representative images of western blot and statistical analysis of TLR7 expression in myocardial tissue (n = 6). *P* = .0008, analyzed by *t*‐test. B, qPCR analysis of TLR7 mRNA level in myocardial tissue (n = 4). Normalized to GAPDH. *P* < .0001, analyzed by *t*‐test. C, Representative images of western blot and statistical analysis of TLR7 expression in isolated AMCMs (n = 6). *P* = .0357, analyzed by *t*‐test. D, qPCR analysis of TLR7 mRNA level in isolated AMCMs (n = 4). Normalized to β‐Actin. *P* = .0237, analyzed by *t*‐test. E, Immunofluorescence double‐labeling with TLR7 and α‐actin after NRVMs were stimulated with LPS (10 µg/mL) or PBS for 24 hours (n = 16‐25cells). Scale bar: 20 µm. F, Representative images of western blot and statistical analysis of TLR7 expression in NRVMs in response to LPS or PBS (n = 6). *P* = .0007, analyzed by *t*‐test. G, qPCR analysis of TLR7 mRNA level in NRVMs in response to LPS or PBS (n = 4). *P* = .0083, analyzed by *t*‐test. The data are shown as the mean ± SEM, with each data point representing a sample of cells or a mouse *P < .05. **P < .01. ***P < .001.

To validate the expression of TLR7 in septic cardiomyopathy, we investigated the expression of TLR7 in the mouse models of sepsis and NRVMs in response to LPS. In vivo, we detected the level of TLR7 in myocardial tissue of mice subjected to LPS injection and found that both TLR7 protein and mRNA expression levels were enhanced compared to mice administrated with saline (Figures 1A and 1B). Subsequently, we assessed the TLR7 expression of AMCMs and found that the TLR7 mRNA and protein levels in AMCMs were slightly increased in mice subjected to LPS (Figures 1C and 1D). Additionally, immunofluorescence of the NRVMs revealed an augmented TLR7 expression in response to LPS (Figure 1E). Furthermore, the results of western blot and qPCR indicated that TLR7 expression was higher in NRVMs following 24 hours LPS administration compared to that in NRVMs subjected to PBS (Figures 1F and 1G). To provide a preliminary understanding of the activation of TLR7 in macrophages , western blot and qPCR were performed, which showed enhanced expression levels of TLR7 in isolated peritoneal macrophages after pretreatment with LPS, as well as the upregulation of HMGB1 (Figures S1B and S1C). On the other hand, LPS contributed to macrophages injury, and the broken DNA was released from the cells (Figures S1D and S1E), which might have resulted in the activation of TLR7 in cardiomyocytes. Additionally, we assessed the levels of TLR4 in the heart tissues of mice with LPS and found that LPS promoted the expression of TLR4 (Figures S1F and S1G)

### 3.2 TLR7 deficiency aggravates cardiac dysfunction in septic cardiomyopathy

**FIGURE 2:**
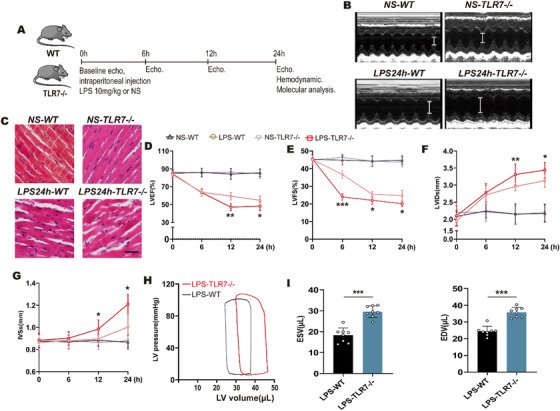
TLR7 deficiency aggravated cardiac dysfunction in septic cardiomyopathy. C57BL/6J WT and TLR7^−/−^ mice were subjected to intraperitoneal injection of LPS (10 mg/kg) or saline. A, Protocol. B, Representative M‐Mode images of the studied groups after LPS administrated for 24 hours (n = 8‐12). White line indicated LVIDs. C, Arrangement of cardiomyocytes was assessed by hematoxylin‐eosin (HE) staining when LPS stimulated for 24 hours (n = 3‐4). Scale bar: 10 µm. D‐G, Echocardiographic examination was used to evaluate cardiac function when LPS administrated for 6, 12, and 24 hours (n = 8‐10). D, Left ventricular ejection fraction (LVEF), *P* = .0072 at 12 hours and *P* = .0248 at 24 hours (vs WT‐LPS group), analyzed by *t*‐test. E, Left ventricle fractional shortening (LVFS), *P* = .0006 at 6 hours, *P* = .0316 at 12 hours and *P* = .0248 at 24 hours (vs WT‐LPS group), analyzed by *t*‐test. F, Left ventricular internal diameter at systole (LVIDs), *P* = .0069 at 12 hours and *P* = .0231 at 24 hours (vs WT‐LPS group), analyzed by *t*‐test, and G, interventricular septal systolic thickness (IVSs) were calculated from echocardiography. *P* = .0329 at 12 hours and *P* = .0274 at 24 hours (vs WT‐LPS group), analyzed by *t*‐test. H and I, Pressure‐volume (PV) loop measurements were recorded when LPS stimulation for 24 hours. H, Representative PV loops of WT and TLR7^−/−^ mice in response LPS, and I, analysis of end systolic volume (ESV) and end diastolic volume (EDV) (n = 7‐10). *P* = .0005 in ESV and *P* = .0003 in EDV (vs WT‐LPS group), analyzed by *t*‐test. The data are shown as the mean ± SEM, with each data point representing a mouse *P < .05. **P < .01. ***P < .001.

To explore the potential effects of TLR7 in septic cardiomyopathy, we first detected cardiac function in TLR7^−/−^ and WT mice challenged with LPS by echocardiography and hemodynamics (Figure 2A). As shown in Figure 2B, LPS injection contributed to the systolic dysfunction, and TLR7 knockout further exacerbated this adverse effect under LPS treatment. Furthermore, there was a regular arrangement of myocardial fibers in saline‐WT and saline‐TLR7 KO mice, and LPS injection led to randomly and loosely arranged myocardial fibers, whereas TLR7 deficiency promoted LPS‐induced cardiomyocytes disarray in the heart section, as identified by H&E staining (Figure 2C). Echocardiography was recorded before LPS stimulation and at 6, 12, and 24 hours after LPS stimulation to assess systolic function (Figures 2D‐2G). As expected, systolic function (evaluated based on EF) decreased in WT mice after LPS stimulation for 24 hours. Notably, TLR7^−/−^ mice showed further reduced EF (Figure 2D). Decreased FS in TLR7^−/−^ mice after LPS injection (Figure 2E) and increased LV internal diameter at systole (Figure 2F) were observed. Furthermore, interventricular septum thickness at end‐systole increased in TLR7^−/−^ mice compared with WT mice (Figure 2G).The end‐systolic volume in TLR7^−/−^ mice was significantly higher compared with that in WT mice, along with a reduction in cardiac output in TLR7^−/−^ mice (table. S3), indicating a lower level of emptying of LV volume, while the EDV in the TLR7^−/−^ mice was increased, indicating an enlargement in LV dilation (Figures 2H and 2I).

### 3.3 Myocyte size, cardiac inflammation, and apoptosis are unchanged in TLR7 deficiency mice

**FIGURE 3:**
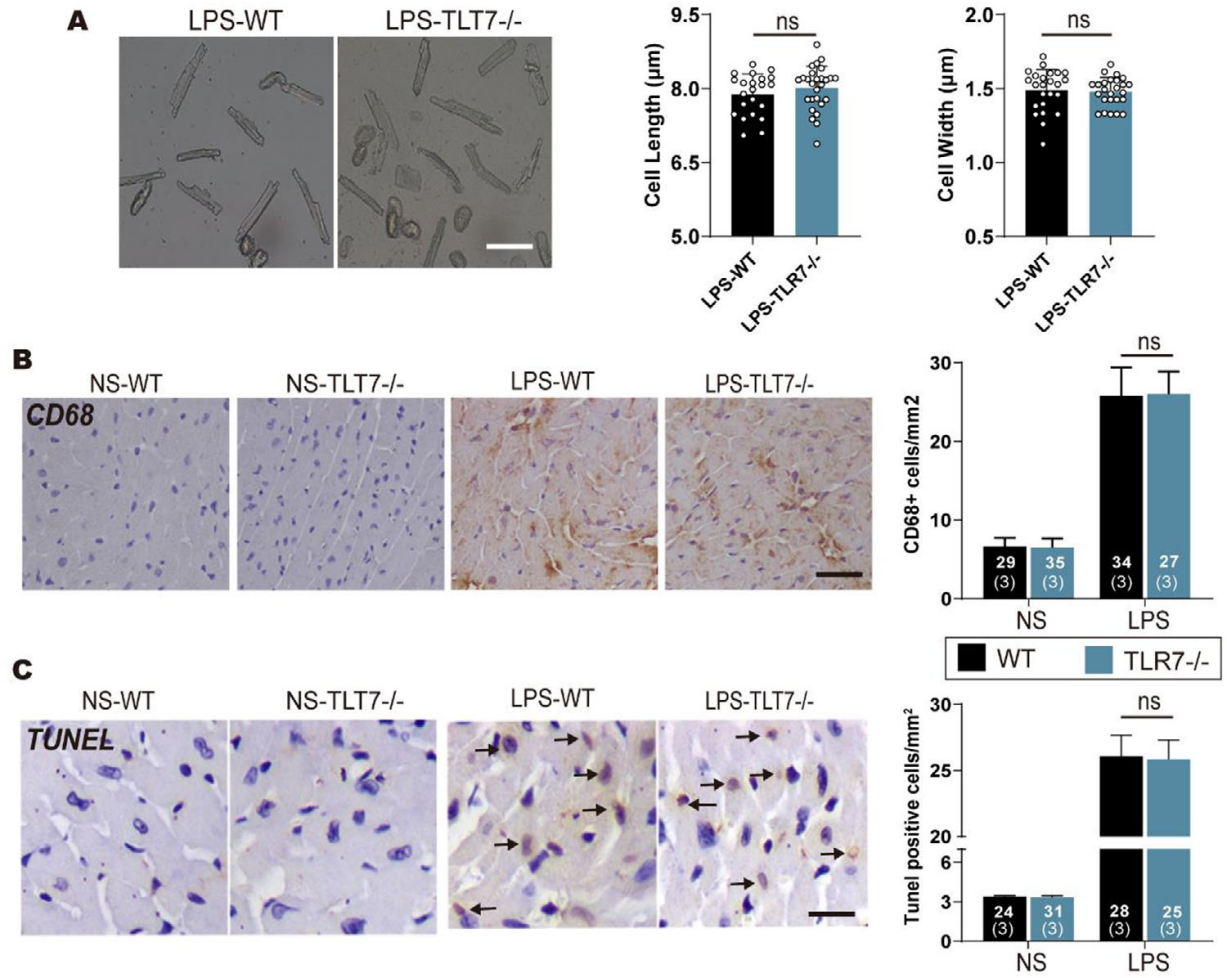
Myocyte size, cardiac inflammation, and apoptosis were unchanged in TLR7 deficiency mice. AMCMs were isolated from WT and TLR7−/− mice subjected to disposable intraperitoneal injection of LPS (10 mg/kg) for 24 hours. A, Representative morphology of AMCMs, and cell length and width were detected (n = 20‐30 cells). Scale bar: 10 µm. *P* = .8274 in length and *P* = .9274 in width, analyzed by one‐way ANOVA with the Bonferroni post‐hoc test. B, Representative images of immunohistochemistry and quantification of CD68 (n = 27‐35 from 3 mice). Scale bar: 120 µm. *P* = .7189, analyzed by one‐way ANOVA with the Bonferroni post‐hoc test. C, Representative images of TUNEL (TdT mediated dUTP Nick End Labeling) and statistical analysis, and the black arrow indicated positive cell (n = 24‐31 from three mice). Scale bar: 50 µm. *P* = .8817, analyzed by one‐way ANOVA with the Bonferroni post‐hoc test. The data are shown as the mean ± SEM, with each data point representing a cell or a mouse Abbreviation: ns, no significance.

Adequate data for dysregulated cardiac Ca2+ handling were available in a case involving abnormal hypertrophic growth.^28^, ^29^, ^30^ To confirm whether TLR7 deficiency resulted in anomalies in myocyte size, we detected cell length and width in isolated AMCMs. As shown in Figure 3A, there was no difference in cell length and mean width in any of the groups. Given the role of TLR7 in immunoregulation and cell death,^31^, ^32^ we determined the effects of TLR7 on LPS‐associated inflammation and apoptosis. Immunoblot analysis revealed that HMGB1 and iNOS proteins increased in response to LPS, while there was no significant difference between WT and TLR7^−/−^ mice (Figure S2A). Consistently, LPS promoted the levels of the pro‐inflammatory factory in serum, but there was no difference between WT and TLR7‐/‐ mice in response to LPS (Figure S2B). Furthermore, immunohistochemistry and immunofluorescence analysis indicated enhanced CD68 and CD4 expression in mice challenged with LPS, and TLR7 deficiency did not affect the inflammatory cell infiltration in the heart (Figures 3D and 3E). Besides, the mRNA level of inflammatory‐related factors such as IL‐1β, IL‐6, TNFα, and MCP‐1 was monitored in cardiac lysates from LPS induced WT and TLR7^−/−^ mice for 24 hours. qPCR results showed that TLR7 knockout resulted in limited impact on LPS‐induced inflammation (Figure S2C). Subsequently, we investigated the LPS‐induced cardiomyocytes apoptosis. Western blot analysis indicated that the level of cleaved caspase‐3 and Bax were upregulated in heart lysates in response to LPS, while there was no difference in the expression between WT and TLR7^−/−^ mice (Figure S2F). Moreover, the examination of caspase‐3 activity indicated the minimal difference between WT and TLR7^−/−^ mice which were attributed to sepsis (Figure S2G). Simultaneously, immunohistochemistry results revealed that LPS injection promoted apoptosis in the heart, while TLR7 knockout did not change the level of TUNEL positive cells (Figure 3C), implying that TLR7 deficiency rarely affected apoptosis following LPS stimulation for 24 hours. Subsequently, we accessed oxidative stress in heart tissues and found that LPS injection contributed to oxidative stress in the heart, while there was no difference between TLR7 depletion and WT mice treated with LPS (Figures S2H and S2I). Besides, the 7‐day percent survival in the NS + WT and NS + TLR7 KO groups was almost 100%. However, 7 days after LPS injection, there was a significant decline in the percent survival in the LPS + WT group (vs NS + WT group). With the TLR7 knockout in mice, the percent survival in the LPS + TLR7 KO group further decreased (vs LPS + WT group), as shown in supplementary Figure 2J.

### 3.4 TLR7 deficiency mice show a decreased Ca^2+^ transient in response to sepsis

**FIGURE 4:**
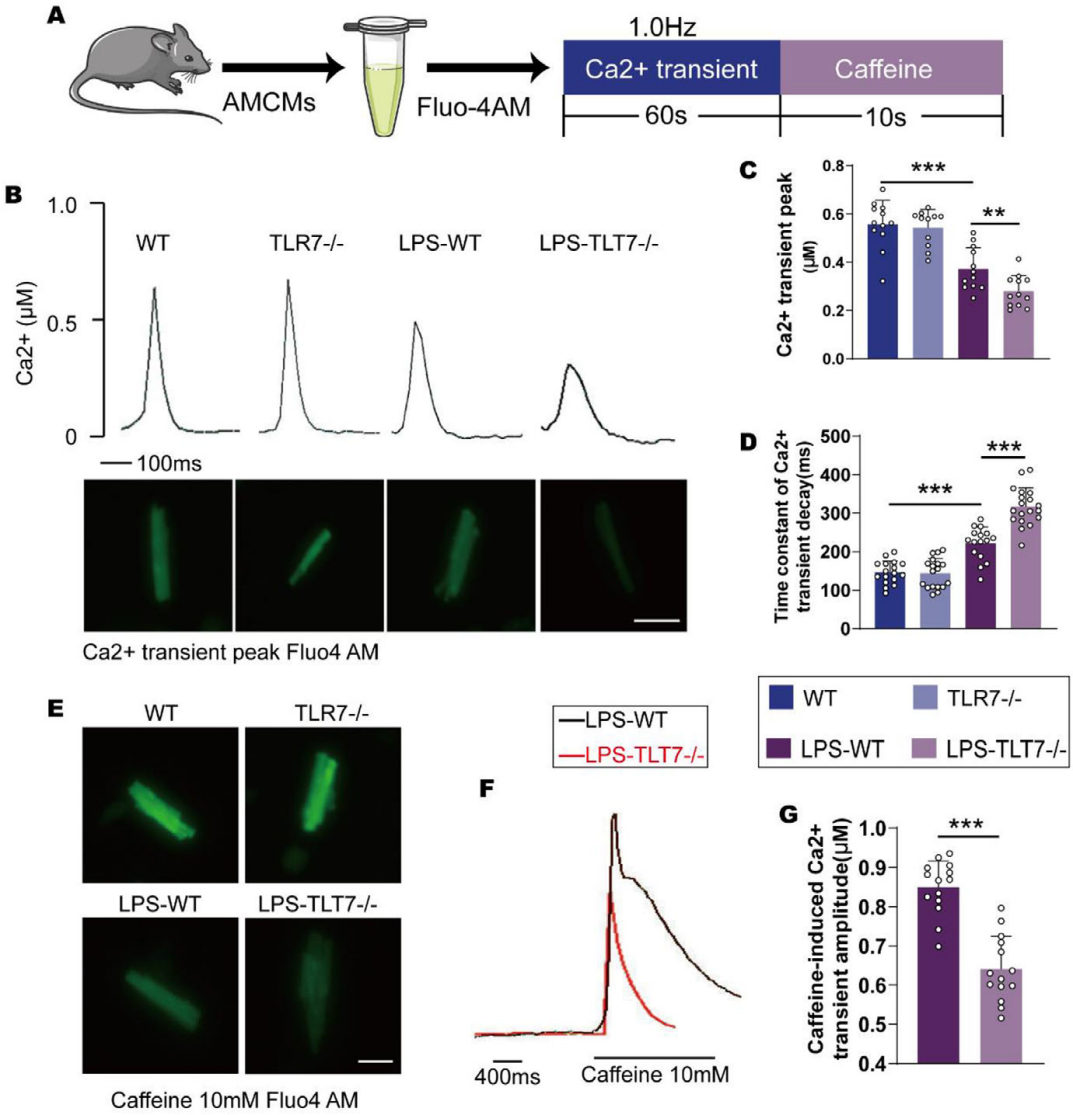
TLR7 deficiency exacerbated sepsis‐induced Ca^2+^ handling disorder in cardiomyocytes. Cardiomyocytes isolated from WT and TLR7^−/−^ mice administrated with LPS or saline were incubated with Fluo‐4 AM to detected Ca^2+^ handling. A, Protocol. B, Representative Ca^2+^ transient curve and (lower panel) Fluo‐4 fluorescence of Ca^2+^ transient peak (n = 10‐14). Scale bar: 20 µm. C, Ca^2+^‐transient amplitudes quantification of AMCMs in indicated groups (n = 10‐14). *P* = .0007 (vs NS‐WT) and *P* = .0052 (vs LPS‐WT), analyzed by one‐way ANOVA with the Bonferroni post‐hoc test. F = 30.72. D, Decay of Ca^2+^ transient was quantified (n = 15‐20). E‐G, the level of Ca^2+^ in the sarcoplasmic reticulum was detected by pretreated with caffeine (10 mM). *P* = .0004 (vs NS‐WT) and *P* = .0009 (vs LPS‐WT), analyzed by one‐way ANOVA with the Bonferroni post‐hoc test. F = 79.91. E, Fluo‐4 fluorescence of caffeine‐induced Ca^2+^ transient peak, Scale bar: 20 µm. F, Typical curve, and G, quantification of the caffeine‐induced Ca^2+^ transient peak in indicated groups (n = 10‐15). *P* = .0002, analyzed by *t*‐test. The data are shown as the mean ± SEM, with each data point representing an adult mice cardiomyocyte *P < .05. **P < .01. ***P < .001.

Cardiac dysfunction in patients with septic cardiomyopathy is reported to be frequently accompanied by decreased Ca^2+^ handling in cardiomyocytes.^33^, ^34^ To explore whether TLR7 provided alteration in Ca^2+^ handling, cardiomyocytes isolated from mice and administrated with LPS for 24h were irritated at 1.0 Hz to elicit Ca^2+^ transient (Figure 4A). Interestingly, the Ca^2+^ transient peak (systolic Ca^2+^) in AMCMs extracted from TLR7^−/−^ mice was significantly reduced compared with that of WT mice intraperitoneally injected with LPS (Figures 4B and 4C). Moreover, the time constant of Ca^2+^ transient decay in the cardiomyocytes of TLR7^−/−^ mice was approximately 1.5‐fold that of the WT mice (Figures 4B and 4D). A rapid bolus of 10 mM caffeine was utilized at the end of the process to free all the Ca^2+^ from the SR into the cytosol (Figure 4E), and this enabled the evaluation of SR Ca^2+^ content. The SR Ca^2+^ content of the TLR7^−/−^ mice were found to be less than that in LPS‐treated WT mice (Figures 4F and 4G). This suggested that TLR7 deficient micehad reduced Ca^2+^ transient in response to sepsis.

### 3.5 Ca^2+^ handling disorder in TLR7‐deficient mice is associated with downregulated RyR2 and Serca and reduced Serca activity via PLN dephosphorylation

**FIGURE 5:**
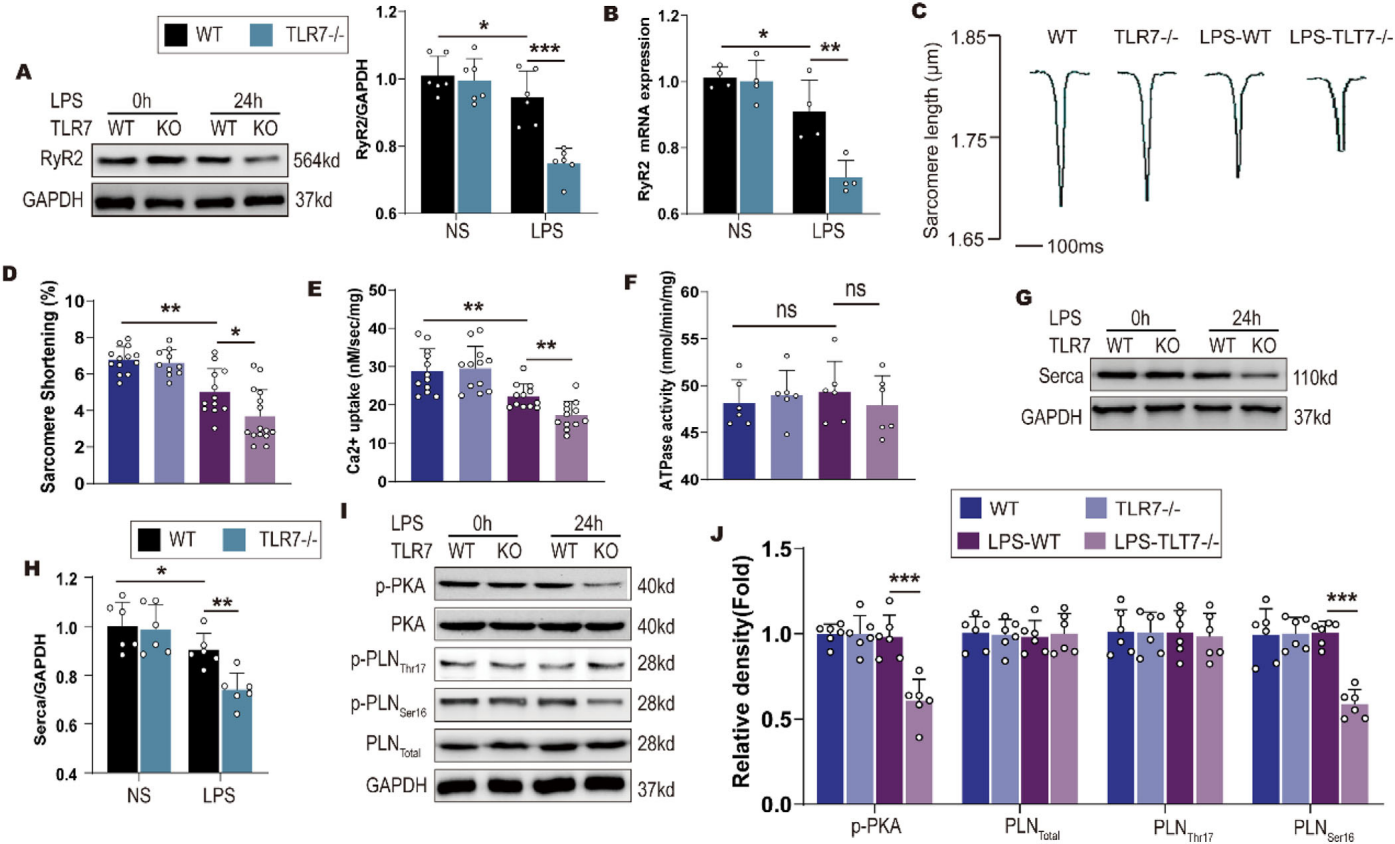
TLR7 knockout aggravated sepsis‐induced downregulated RyR2 and Serca, and reduced Serca activity by phospholamban dephosphorylation. After LPS stimulated for 24 hours, cardiac tissue and AMCMs were harvested from WT and TLR7^−/−^ mice. A, Representative images of western blot and statistical analysis of RyR2 expression in cardiac tissue (n = 6), *P* = .0225 (vs NS‐WT) and *P* = .0005 (vs LPS‐WT), analyzed by two‐way ANOVA with the Bonferroni post‐hoc test. F = 12.38, and B, qPCR analysis of RyR2 mRNA level in cardiac tissue (n = 4), *P* = .0411 (vs NS‐WT) and *P* = .0072 (vs LPS‐WT), analyzed by two‐way ANOVA with the Bonferroni post‐hoc test. F = 8.597. Normalized to GAPDH. C, Representative tracing of sarcomere shortening in AMCMs isolated from indicated mice, and D, accumulated data of sarcomere shortening (n = 10‐15 cells), *P* = .0095 (vs NS‐WT) and P = .0325 (vs LPS‐WT), analyzed by one‐way ANOVA with the Bonferroni post‐hoc test. F = 22.24. E, Ca^2+^ uptake activity of Serca in sarcoplasmic reticulum was analyzed by incubating with Fura 2 (n = 8‐12), *P* = .0052 (vs NS‐WT) and *P* = .0077 (vs LPS‐WT), analyzed by one‐way ANOVA with the Bonferroni post‐hoc test. F = 17.87, and F, ATPase activity of Serca was measured by ATPase activity Kit (n = 6). G and H, Representative images of western blot and statistical analysis of Serca protein expression in cardiac tissue (n = 6), *P* = .0337 (vs NS‐WT) and *P* = .0058 (vs LPS‐WT), analyzed by two‐way ANOVA with the Bonferroni post‐hoc test. F = 6.291. I and J, Representative images of western blot and statistical analysis of PLN, pSer16‐PLN, pThr17‐PLN expression in cardiac tissue (n = 6). *P* = .0008 in p‐PKA and *P* = .0007 in PLN ser16 (vs LPS‐WT), analyzed by one‐way ANOVA with the Bonferroni post‐hoc test. F = 16.34. The data are shown as the mean ± SEM, with each data point representing a cell sample or a mouse Abbreviation: ns, no significance. *P < .05. **P < .01. ***P < .001.

Previous studies have reported that TLR9 regulates the synthesis of mitochondrial ATP by inhibiting Serca expression and modulating Ca^2+^ handling between the SR/ER and mitochondria.^35^ To investigate the mechanism of TLR7 in Ca^2+^ handling, we determined the phosphorylation level of critical Ca^2+^ handling factors involved in the control of Serca‐mediated Ca^2+^ uptake and RyR2‐mediated Ca^2+^ release in isolated cardiomyocytes following LPS stimulation for 24 hours (Figure S3A). The results indicated that the expression of Serca and RyR2 was slightly decreased in WT mice subjected to LPS injection. However, both Serca and RyR2 in TLR7^−/−^ mice were greatly reduced than in WT mice (Figures 5A, 5G, and 5H). Immunofluorescence and qPCR showed that TLR7 deficiency reduced the expression of Serca and RyR2 in response to sepsis (Figure 5B, Figures S3B and S3C). Subsequently, we further examined systolic function which was determined by sarcomere shortening and found that TLR7 knockout aggravated the decline of sarcomere shortening in WT mice challenged with LPS (Figures 5C and 5D). Furthermore, compared with the WT mice, TLR7 deficiency inhibited Ca^2+^ uptake but not ATPase activity of Serca (Figures 5E and 5F), and reduced cAMP activity when pretreated with LPS (Figure S3D). Western blot analysis indicated that the expression of phosphorylated PLN in Serine 16 instead of Threonine 17 decreased in TLR7^−/−^ mice in response to sepsis compared with WT mice, along with downregulation of phosphorylated PKA (Figures 5I and 5J). However, Ca^2+^ handling‐related proteins such as CaMKII and Stim‐1 were not altered in both WT and TLR7^−/−^ mice in response to LPS stimulation (Figure S3E). Immunoprecipitation revealed that PKA directly interacted with PLN, which interacted with Serca to regulate the function of Serca in cardiomyocytes (Figures S3F and S3G). To justify that TLR7 signals through cAMP, WT, or TLR7^−/−^ AMCMs were pretreated with TLR7‐specific agonist (loxoribine) and WT AMCMs incubated with an antagonist (IRS661). The cAMP activity and levels of pro‐inflammatory factors in isolated AMCMs and peritoneal macrophages were measured (Figure S4A). Loxoribine did not affect the expression of pro‐inflammatory factors in cardiomyocytes (Figures S4B and S4C), whereas loxoribine promoted the production of TNFα and IL‐1β in peritoneal macrophages (Figures S4F and S4G). The cAMP activity was also found to be significantly enhanced in response to loxoribine but showed no effect in TLR7^−/−^ AMCMs (Figure S4D). Consistently, TLR7 inhibitor decreased cAMP activity in response to LPS (Figure S4E). The effects of loxoribine in vivo were confirmed (Figure S4H). As expected, TLR7 agonist was found to alleviate LPS‐induced cardiac dysfunction in mice (Figures S4I and S4J).

### 3.6 TLR7 plays a critical role in cultured neonatal ventricular myocytes (NRVMs) in response to LPS by impacting Ca^2+^ handling in vitro

**FIGURE 6:**
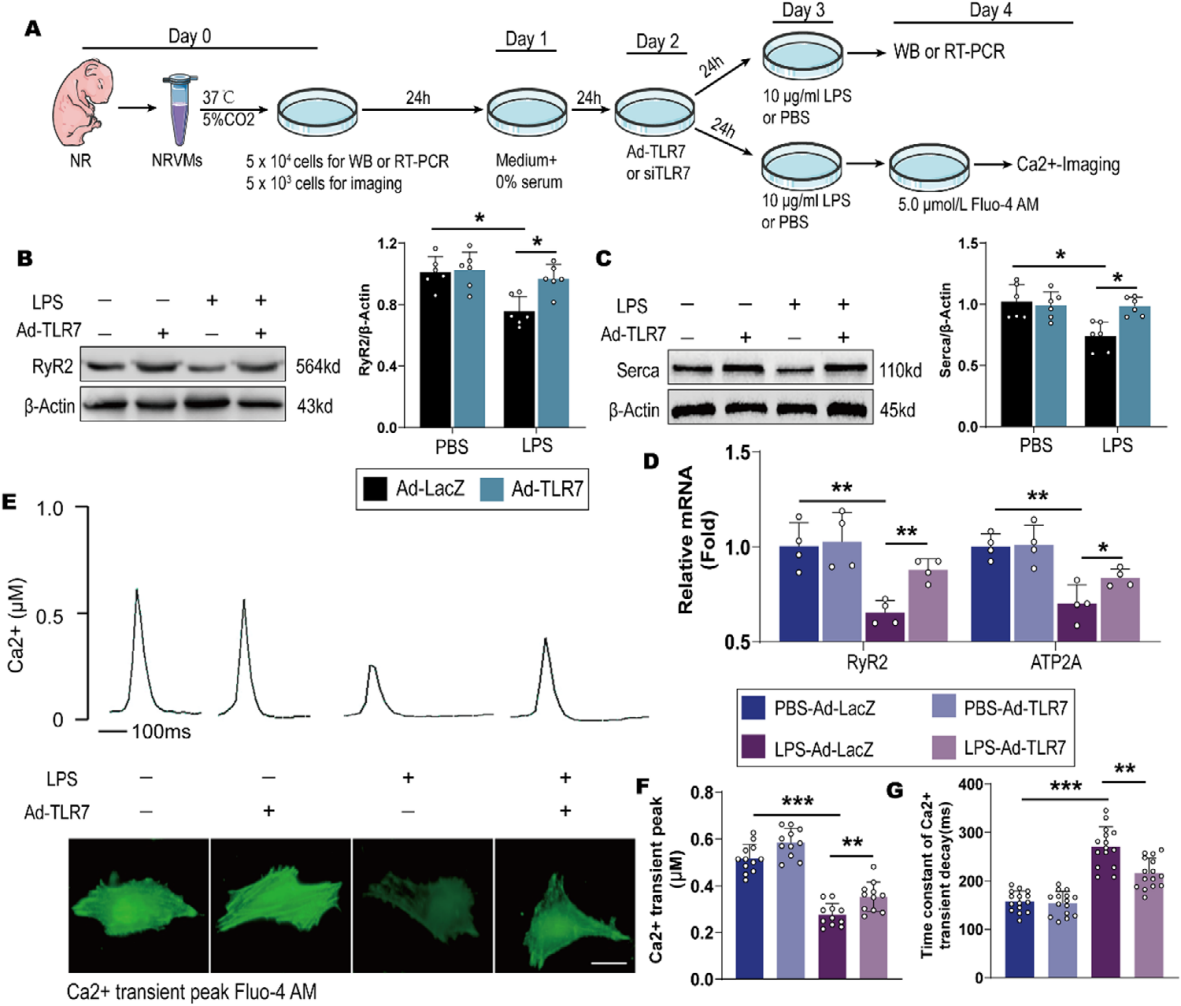
Effect of TLR7 on Ca^2+^ handling in cultured neonatal rat ventricular myocytes. Neonatal rat ventricular myocytes (NRVMs) transfected with Ad‐TLR7 or Ad‐LacZ were stimulated with LPS (10 µg/mL) or PBS for 24 hours. A, Protocol showing the method of neonatal rat ventricular myocytes (NRVMs) culture. B, Representative images of western blot and statistical analysis of RyR2 expression (n = 6), *P* = .0276 (vs PBS‐Ad‐LacZ) and *P* = .0269 (vs LPS‐Ad‐LacZ), analyzed by two‐way ANOVA with the Bonferroni post‐hoc test. F = 7.385. C, Representative images of western blot and statistical analysis of Serca expression (n = 6), *P* = .0382 (vs PBS‐Ad‐LacZ) and *P* = .0274 (vs LPS‐Ad‐LacZ), analyzed by two‐way ANOVA with the Bonferroni post‐hoc test. F = 8.798. D, qPCR analysis of mRNA level for the gene coding for Serca (ATP2A2) and RyR2 (n = 3‐4). Normalized to β‐Actin. For RyR2 mRNA, *P* = .0064 (vs PBS‐Ad‐LacZ) and *P* = .0092 (vs LPS‐Ad‐LacZ), analyzed by one‐way ANOVA with the Bonferroni post‐hoc test. F = 8.527. For SERCA mRNA, *P* = .0037 (vs PBS‐Ad‐LacZ) and *P* = .031 (vs LPS‐Ad‐LacZ), analyzed by one‐way ANOVA with the Bonferroni post‐hoc test. F = 6.725. E, Typical Ca^2+^ transient curve and (lower panel) Fluo‐4 fluorescence of Ca^2+^ transient peak (n = 10‐14). Scale bar: 20 µm. F, Quantification of Ca^2+^‐transient amplitude in indicated NRVMs (n = 10‐14), *P* = .0008 (vs PBS‐Ad‐LacZ) and *P* = .0055 (vs LPS‐Ad‐LacZ), analyzed by two‐way ANOVA with the Bonferroni post‐hoc test. F = 11.428. G, Decay of Ca^2+^ transient was quantified (n = 10‐14), *P* = .0003 (vs PBS‐Ad‐LacZ) and *P* = .0082 (vs LPS‐Ad‐LacZ), analyzed by two‐way ANOVA with the Bonferroni post‐hoc test. F = 22.362. The data are shown as the mean ± SEM, with each data point representing a NRVM or a cell sample *P < .05. **P < .01. ***P < .001.

To further investigate the role of TLR7 in vitro, we transfected NRVMs with adenoviral vectors loaded with TLR7 (Ad‐TLR7) and Ad‐LacZ or TLR7 siRNA oligos and control siRNA for 24 hours (Figures S5A and S5B). LPS was incubated with NRVMs for 24 hours as previously described.^36^ The effect of TLR7 knockdown and LPS on Ca^2+^ handling was investigated in vitro (Figure 6A). Immunoblotting analysis revealed that overexpression of TLR7 restored LPS‐induced reduction of RyR2 and Serca (Figures 6B and 6C), while TLR7 knockdown showed an opposite effect (Figures S5C and S5E). qPCR analysis showed that genes coding for Serca (ATP2A2) and RyR2 were inhibited in NRVMs pretreated with LPS, and TLR7 overexpression remarkably restored the reduction of RyR2 and Serca (Figure 6D). However, TLR7 knockdown showed opposite outcomes in NRVMs pretreated with LPS (Figures S5D and S5F). Subsequently, to explore whether TLR7 caused anomaly in Ca^2+^ handling in vitro, NRVMs incubated with LPS were stimulated at 1.0 Hz to elicit Ca^2+^ transient. Notably, LPS administration showed a lower Ca^2+^ transient peak and prolonged‐time constant of Ca^2+^ transient decay. Nonetheless, TLR7 overexpression ameliorated LPS‐induced disorder of Ca^2+^ transient in NRVMs (Figures 6E‐6G).

### 3.7 Cardiac‐specific overexpression of TLR7 alleviates sepsis‐induced cardiac dysfunction

**FIGURE 7:**
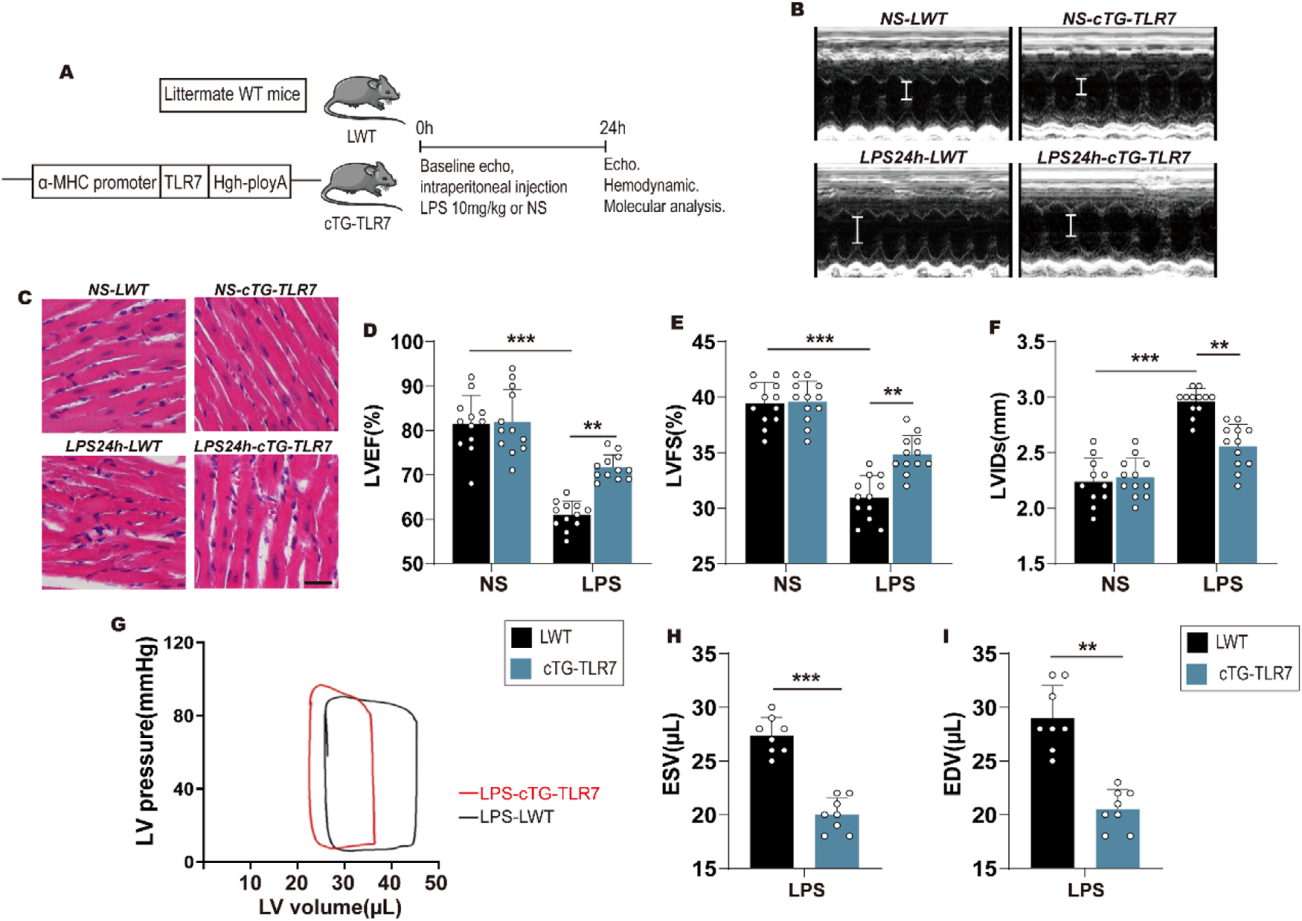
Cardiac‐specific overexpression of TLR7 alleviated sepsis‐induced cardiac dysfunction. A, Protocol and schematics of TLR7 transgenic construct. B, Representative M‐Mode images of the studied groups after LPS administrated for 24h (n = 10‐12). C, Representative HE‐stained heart sections from littermate WT (LWT) and cardiac‐specific overexpression TLR7 gene (cTG‐TLR7) mice pretreated with or without LPS stimulated for 24 hours (n = 3‐4). Scale bar: 50 µm. D‐F, Echocardiographic examination was used to evaluate cardiac function when LPS administrated for 24 hours (n = 10‐12). D, Left ventricular ejection fraction, *P* < .0001 (vs NS‐LWT) and *P* = .0041 (vs LPS‐LWT), analyzed by two‐way ANOVA with the Bonferroni post‐hoc test. F = 11.35, E, left ventricle fractional shortening (LVFS), *P* < .0001 (vs NS‐LWT) and *P* = .0067 (vs LPS‐LWT), analyzed by two‐way ANOVA with the Bonferroni post‐hoc test. F = 12.02, and F, left ventricular end systolic diameter (LVIDs) were calculated from echocardiography, *P* < .0001 (vs NS‐LWT) and *P* = .0074 (vs LPS‐LWT), analyzed by two‐way ANOVA with the Bonferroni post‐hoc test. F = 17.76. G‐I, Pressure‐volume (PV) loop measurements were recorded when LPS stimulation for 24 hours. G, Representative PV loops of LWT and cTG‐TLR7 mice in response LPS, and H and I, analysis of end systolic volume (ESV) and end diastolic volume (EDV) (n = 8‐10), *P* = .0003 in ESV and *P* = .0085 in EDV, analyzed by *t*‐test. The data are shown as the mean ± SEM, with each data point representing a mouse *P < .05. **P < .01. ***P < .001.

To further investigate the effect of TLR7 on septic cardiomyopathy, we generated cardiac‐specific TLR7‐transgenic (cTG‐TLR7) mice, subjected to LPS for 24 hours, and littermates wild‐type (LWT) mice were used as a control (Figure 7A). As shown in Figure 7B, compared to the LWT mice, cTG‐TLR7 alleviated sepsis‐induced systolic function in vivo. Moreover, H&E staining showed that cTG‐TLR7 relieved LPS‐induced cardiomyocytes disarray in the heart (Figure 7C). Simultaneously, systolic function (evaluated based on EF) deteriorated in LWT mice after LPS stimulation for 24 hours. However, cTG‐TLR7 mice restored the LPS‐induced reduction of EF compared to LWT mice (Figure 7D). The enhanced FS in cTG‐TLR7 mice after LPS injection (Figure 7E) was due to improved cardiac function, as shown by the smaller left ventricle internal diameter tested at systole (Figure 7F). A reduction in IVSs was also observed in cTG‐TLR7 mice compared to LWT mice in response to sepsis (Figure S6A). Besides, compared with LWT mice, the ESV in cTG‐TLR7 mice decreased, while the cardiac output in cTG‐TLR7 mice increased (table. S4), implying a higher‐level emptying of LV volume. The EDV in the cTG‐TLR7 mice decreased, indicating a reduction in LV dilation (Figures 7G‐7I). cTG‐TLR7 inhibited LPS‐induced reduction of cAMP activity compared with LWT mice (Figure S6B). At the molecular level, western blot analysis indicated that overexpression of TLR7 promoted the production of phosphorylated PKA and increased the expression of phosphorylated PLN in Serine 16 without any change in the total PLN level in heart tissues (Figures S6C‐S6F).

### 3.8 Inhibition of PKA abolishes TLR7's cardio‐protective effects in response to sepsis in vivo

**FIGURE 8:**
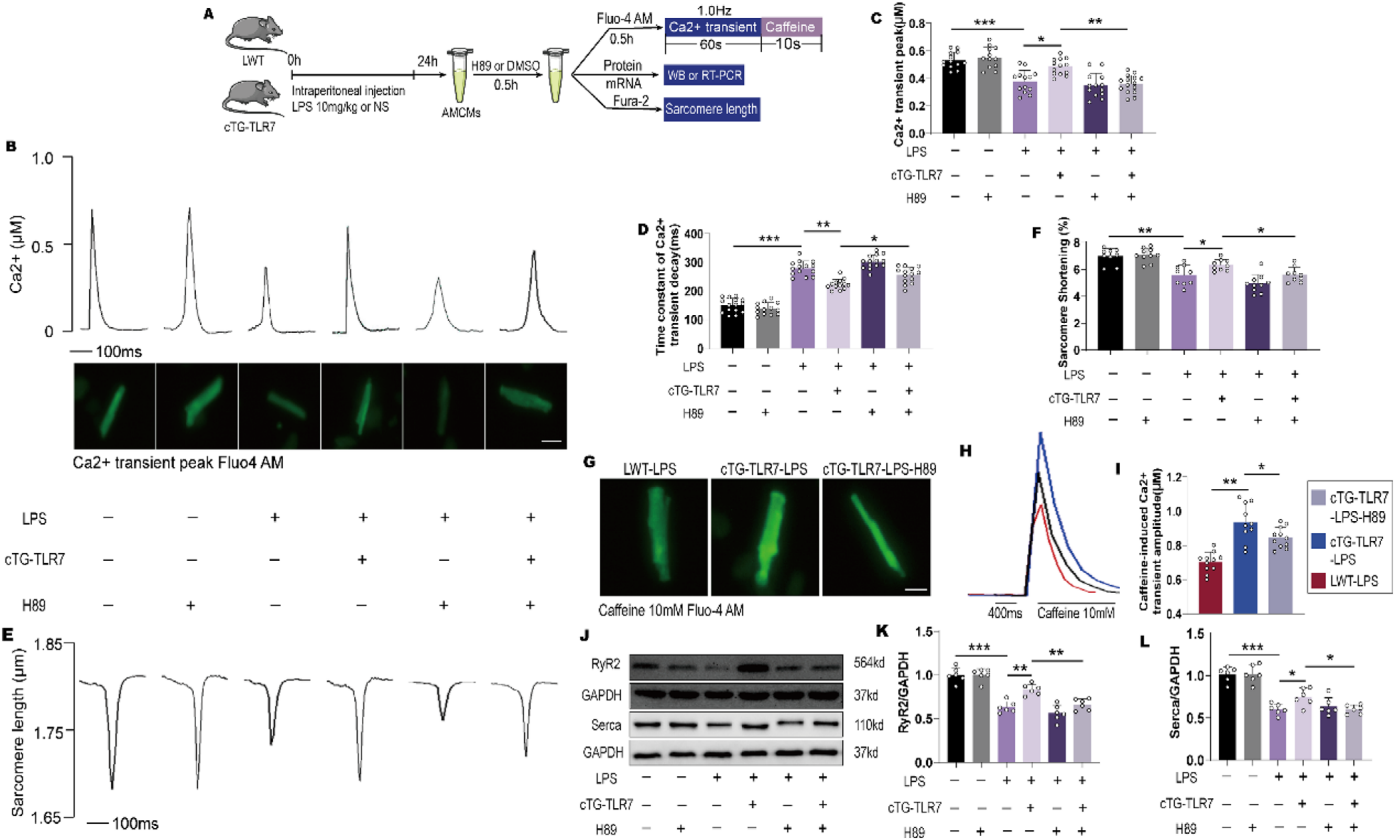
Inhibition of PKA hindered TLR7's cardioprotective effects in response to sepsis. A, Protocol. B, Typical Ca^2+^ transient curve and (lower panel) Fluo‐4 fluorescence of Ca^2+^ transient peak (n = 10‐14). Scale bar: 20 µm. C, Ca^2+^‐transient amplitudes quantification in AMCMs from LWT and cTG‐TLR7 mice in indicated groups (n = 10‐15), *P* = .0007 (vs NS‐LWT‐DMSO), *P* = .0328 (vs LPS‐LWT‐DMSO) and *P* = .0078 (vs LPS‐cTG‐DMSO) analyzed by one‐way ANOVA with the Bonferroni post‐hoc test. F = 21.03. D, Decay of Ca^2+^ transient was quantified (n = 10‐15), *P* = .0004 (vs NS‐LWT‐DMSO), *P* = .0084 (vs LPS‐LWT‐DMSO) and *P* = .0388 (vs LPS‐cTG‐DMSO) analyzed by one‐way ANOVA with the Bonferroni post‐hoc test. F = 36.73. E, Representative tracing of sarcomere shortening from in AMCMs isolated from indicated mice, and F, accumulated data of sarcomere shortening (n = 9‐12), *P* = .0065 (vs NS‐LWT‐DMSO), *P* = .0157 (vs LPS‐LWT‐DMSO) and *P* = .0205 (vs LPS‐cTG‐DMSO) analyzed by one‐way ANOVA with the Bonferroni post‐hoc test. F = 33.84. G‐I, The level of Ca^2+^ in the sarcoplasmic reticulum was detected by pretreated with caffeine (10 mM). G, Fluo‐4 fluorescence of caffeine‐induced Ca^2+^ transient peak, H, typical curve, and I, quantification of the caffeine‐induced Ca^2+^ transient peak (n = 9‐12), *P* = .0053 (vs LWT‐LPS) and *P* = .0162 (vs cTG‐LPS), analyzed by *t*‐test. Scale bar: 20 µm. J‐L, Representative images of western blot and statistical analysis of Serca and RyR2 expression (n = 6). The data are shown as the mean ± SEM, with each data point representing a mouse or a cell *P < .05. **P < .01. ***P < .001.

Previous studies have reported that TLR7 agonists inhibit tumor migration by activating PKA and promoting glioma‐associated oncogene (GLI) phosphorylation.^37^ To explore the role of PKA on TLR7‐mediated cardiac‐protection in septic cardiomyopathy, AMCMs isolated from LWT and cTG‐TLR7 mice were harvested as shown in Figure 8A. AMCMs were pretreated with H89 (a specific PKA inhibitor) or DMSO before Ca^2+^ transient detection. The Ca^2+^ transient peak (systolic Ca^2+^) in AMCMs from cTG‐TLR7 mice was significantly increased compared to that in LWT mice during sepsis, however, the improved Ca^2+^ transient amplitude was blocked by H89 (Figures 8B and 8C). Furthermore, the time constant of Ca^2+^ transient decay in AMCMs isolated from cTG‐TLR7 mice was approximately 70% of that in the LWT mice, yet H89 pretreatment pronged the time constant of Ca^2+^ transient decay (Figures 8B and 8D). Subsequently, we further examined the systolic function, which was measured by sarcomere shortening, and found that cTG‐TLR7 restored the reduction in sarcomere shortening in LWT mice subjected to LPS stimulation. However, H89 pretreatment eliminated the protective effect of cTG‐TLR7 (Figures 8E and 8F). A rapid bolus of 10 mM caffeine was applied at the end of the protocol to assess SR Ca^2+^ content. We found that Ca^2+^ content in AMCMs of the cTG‐TLR7 mice was about 1.5‐fold that in LWT mice, while H89 administration blocked the positive effect of TLR7 overexpression on Ca^2+^ content (Figures 8G‐8I). Western blot analysis showed that H89 inhibited TLR7 overexpression‐mediated upregulation of RyR2 and Serca (Figures 8J‐8L).

## 4 DISCUSSION

In recent years, TLR7 has been intensively studied in virus‐induced immune cell activation and tumor immunity.^38^, ^39^ Besides, TLR7 is a toll‐like receptor that has been recognized as an essential regulator for cardiac dysfunction attributed to myocardial infarction (MI).^16^ To replicate the pathophysiology of sepsis cardiomyopathy, a septic animal model was utilized by administering exogenous LPS intraperitoneally. Herein, we demonstrated that the activation of TLR7 improved sepsis‐induced septic cardiomyopathy. Besides, we also revealed that TLR7 regulated Ca^2+^ handling in the SR/ER by increasing the activity of cAMP, and phosphorylating PLN in Serine 16, which upregulated the level of Ca^2+^ handling‐related proteins such as RyR2 and Serca, and eventually promoted Serca activity in vivo and in vitro. Additionally, we supported the role of TLR7 in regulating cAMP activity using TLR7‐specific agonist (loxoribine) and antagonist (IRS661).

Septic cardiomyopathy was characterized by a large left ventricle and decreased EF.^40^, ^41^, ^42^ Dysfunction of cardiomyocytes Ca2+ handling induced by gram‐negative bacterial outer‐membrane protein (TLR2 ligand), or LPS (TLR4 ligand) has been previously reported.^43^, ^44^ However, the effect of TLR7 remains unknown. Several studies have revealed that TLR7 deficiency reduces the adverse effects of left ventricular remodeling after MI.^16^ In our study, we found that in both myocardial tissues of mice and isolated NRVMs, TLR7 was upregulated in response to LPS. Activation of TLR7 may be attributed to the following mechanisms: (a) Qin et al^45^ reported that outer pathogen associated molecular patterns (LPS) integrates with the inner damage associated molecular pattern (HMGB1), to form a complex that recognizes TLRs in synovial fibroblasts (SFs), hence we detected the HMGB1 level in myocardial tissue. Although TLR4 is the key receptor for LPS to invade cells, there were uncertainties in the activation of TLR4 and the increased levels of HMGB1‐LPS complex in the cell, thereby inducing TLR7 activation; (b) TLR7 is a dual receptor for guanosine and uridine‐rich single‐stranded viral RNA. Perhaps the connection is that during LPS‐insult, cells get damaged, DNA gets released from the cells, thus, increasing extracellular guanosine which is taken up by cardiomyocytes and activated TLR7. Therefore, we detected apoptosis in myocardial tissue; (c) Olivia Majer et al^46^ reported that immune cells release TLR7 into exosomes to suppress autoimmunity, an indication that TLR7 is transported from the immune cells into cardiomyocytes by releasing TLR7 into exosomes. In this study, we only performed a preliminary investigation into the possible mechanism of TLR7 activation, therefore, more evidence needs further research. Besides, activated TLR7 migrates into the Golgi apparatus and endosomes, and enhances the MyD88/IRAK/NF‐κB cascade, ultimately leading to inflammation. However, in this study, no difference in inflammation and apoptosis was reported. Therefore, we deduced that early inflammation associated with LPS was mainly mediated by TLR4, and the role of TLR7 was limited, hence, we focused on the role of TLR7 in cardiomyocytes.

Ca^2+^ enters into or releases from the SR during excitation‐contraction coupling in cardiomyocytes, and unbalanced Ca^2+^ homeostasis leads to cardiac dysfunction and directly results in cardiomyopathy and heart failure.^19^ Some studies report that orphaned RyR2 proteins are associated with HF and RyR2 channels which are assembled into clusters of 10‐20 units along Z‐lines where they are adjoined to L‐type voltage‐gated Ca^2+^ channels, and control Ca^2+^ releases from the SR.^47^, ^48^, ^49^ Several studies have revealed that Serca controls Ca^2+^ uptake into SR/ER, while Serca activity is regulated by PLN, SLN, PP1, and CaMKII.^50^, ^51^, ^52^ Specifically, PLN has been identified to directly interact with Serca and inhibit Ca^2+^ uptake by regulating Serca activity. A recent study has shown that phosphorylation of PLN restores the Serca activity by inhibiting PLN phosphorylation.^52^ Moreover, the phosphorylated site of PLN is mainly located at Serine16 and Threonine17.^50^ PLN is phosphorylated by PKA in vitro, but the mechanism of PKA‐dependent PLN phosphorylation remains unclear, and its significance in cardiac function is controversial.^53^ The current study indicated that the inhibition of PKA abrogated TLR7's cardioprotective effects in response to sepsis. Besides, the results suggested that Serine 16 instead of Threonine 17 of PLN was phosphorylated as a result of cAMP/PKA upregulation in SR/ER. Optimized Ca^2+^ handling was found to be abolished when cardio‐myocytes were subjected to PKA inhibitor. Therefore, we demonstrated that TLR7 protected against sepsis‐induced Ca^2+^ handling by enhancing the cAMP/PKA levels.

Although our previous study suggested that cAMP directly activated PKA in cardiomyocytes,^54^ there is not sufficient evidence of a direct link between cAMP and TLR7, even though TLR7‐induced cytokines may regulate the cAMP activity. RyR2 was found to regulate the release of Ca^2+^ from SR/ER, and previous studies indicate that RyR2 is phosphorylated on serine‐2814 by PLN,^55^ but Ca^2+^ spark was not detected in the current study. Septic cardiomyopathy is characterized by microcirculatory disorders which trigger the chronic microthrombus formation and ultimately result in heart failure. Serca maybe phosphorylated on Threonine 484 and acetylated on Lysine492,^56^, ^57^ thus there is a need to further investigate the modification of Serca in response to sepsis. In this study, the focus was largely on early‐stage sepsis which causes high mortality, and further studies should be conducted for a longer period in the following study.

## CONFLICT OF INTEREST

The authors declare that there is no conflict of interest that could be perceived as prejudicing the impartiality of the research reported.

## Supporting information

Supporting Inforamtion

Supporting Inforamtion
