# Supplementary material for: Activation of Toll‐like receptor 7 provides cardioprotection in septic cardiomyopathy‐induced systolic dysfunction
Source: Clin Transl Med. 2021 Jan 1;11(1):e266. doi: 10.1002/ctm2.266 (PMC7775988; doi:10.1002/ctm2.266)

## Slide 1
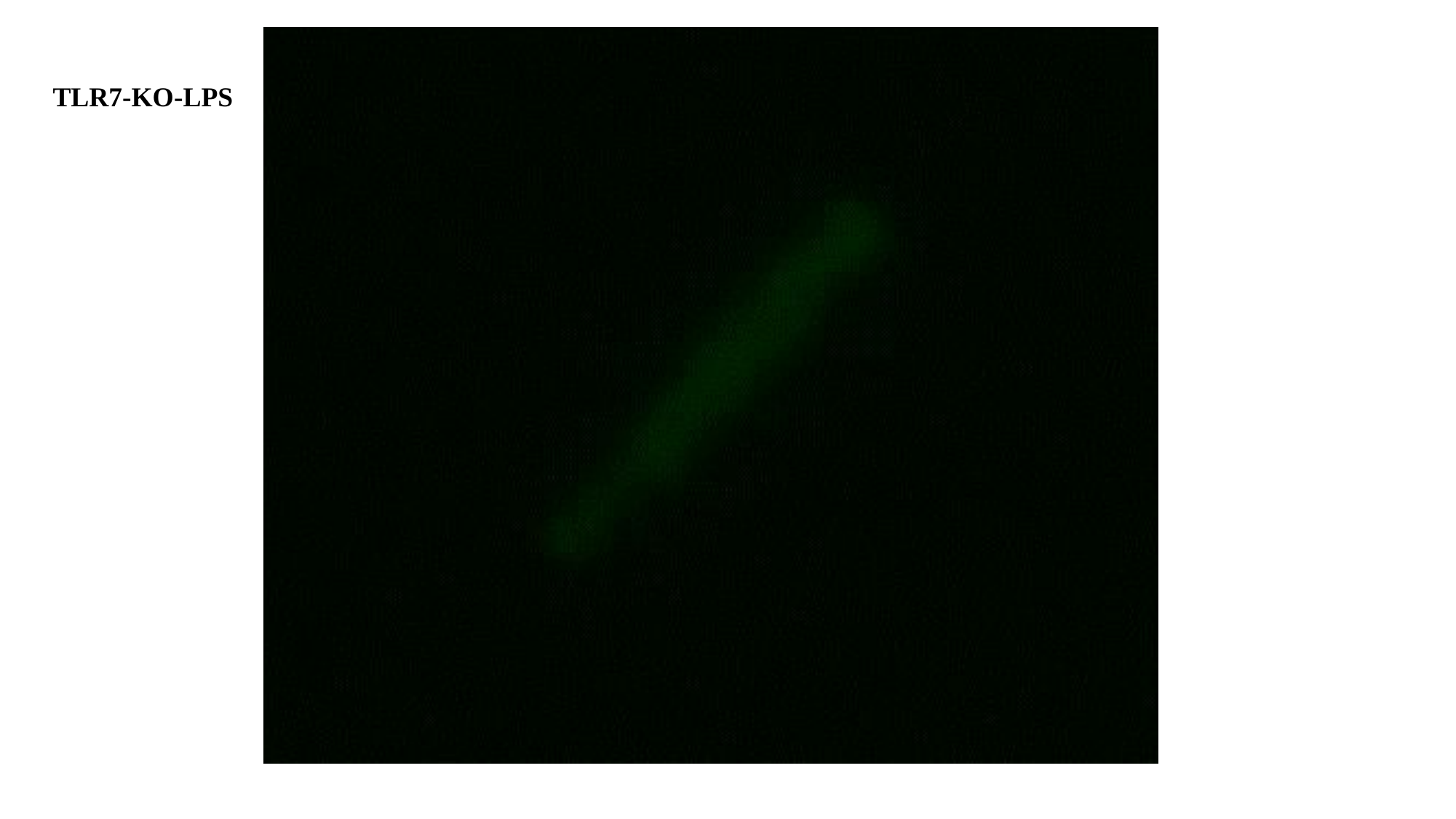

TLR7-KO-LPS

## Slide 2
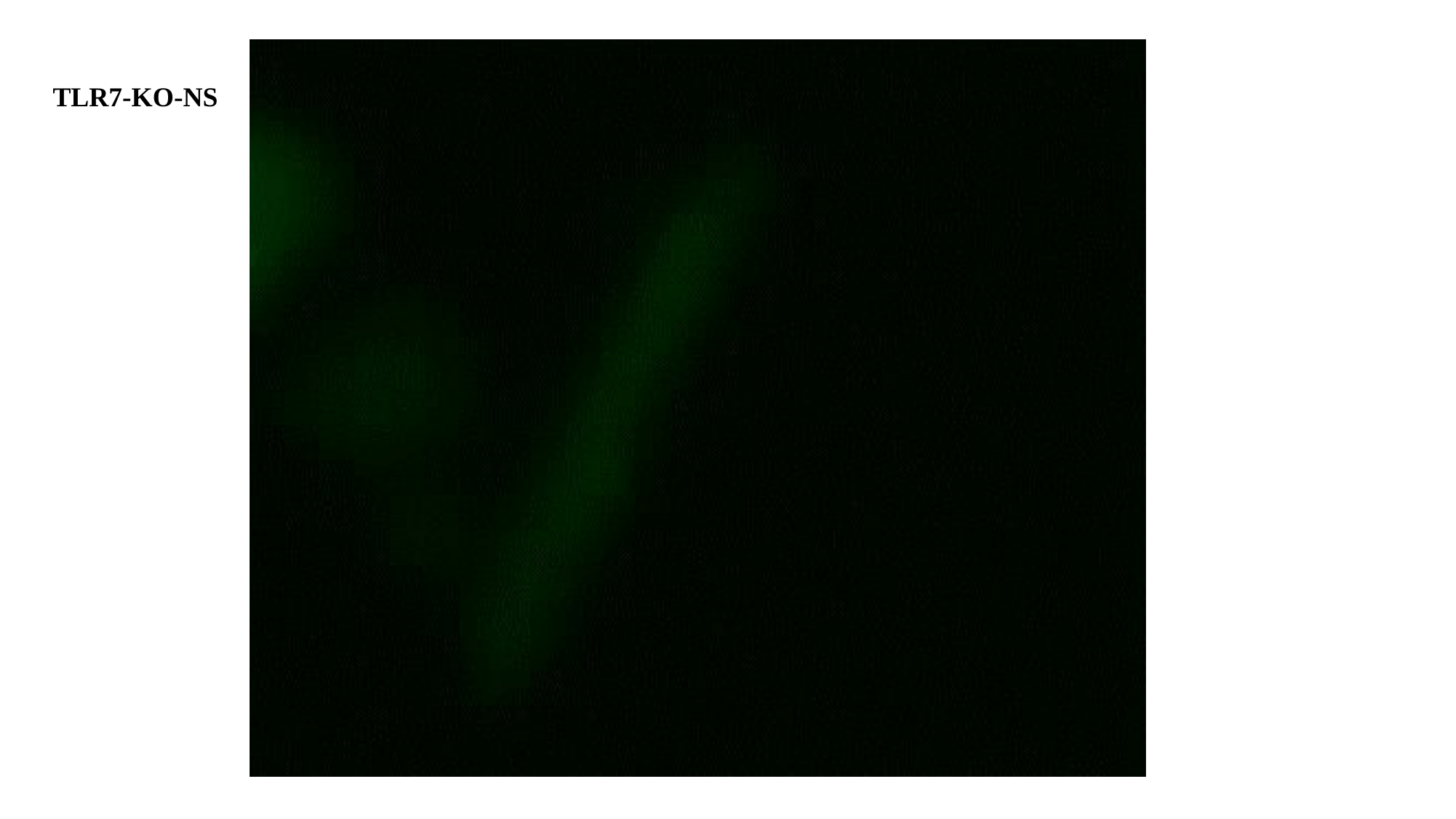

TLR7-KO-NS

## Slide 3
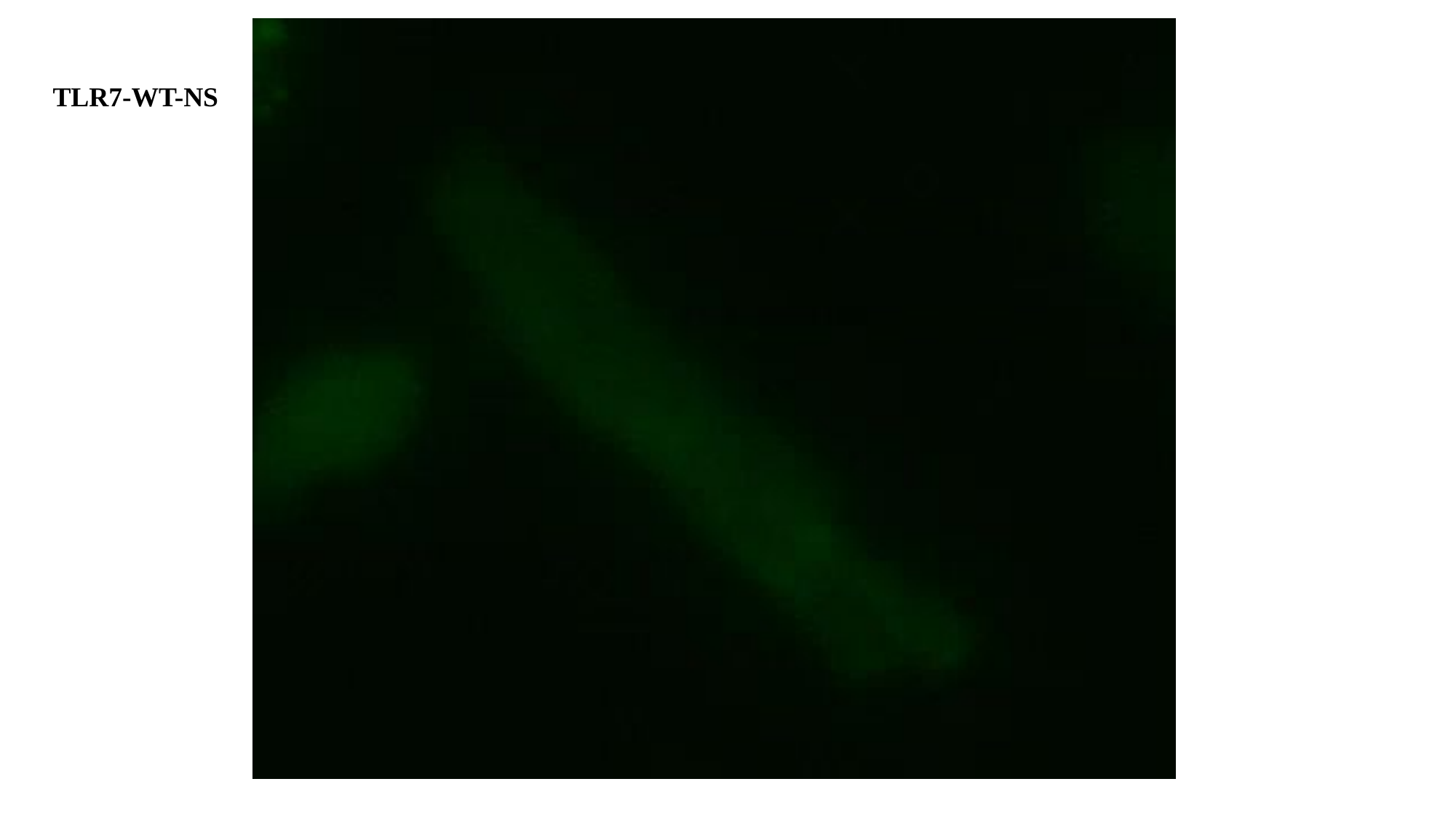

TLR7-WT-NS

## Slide 4
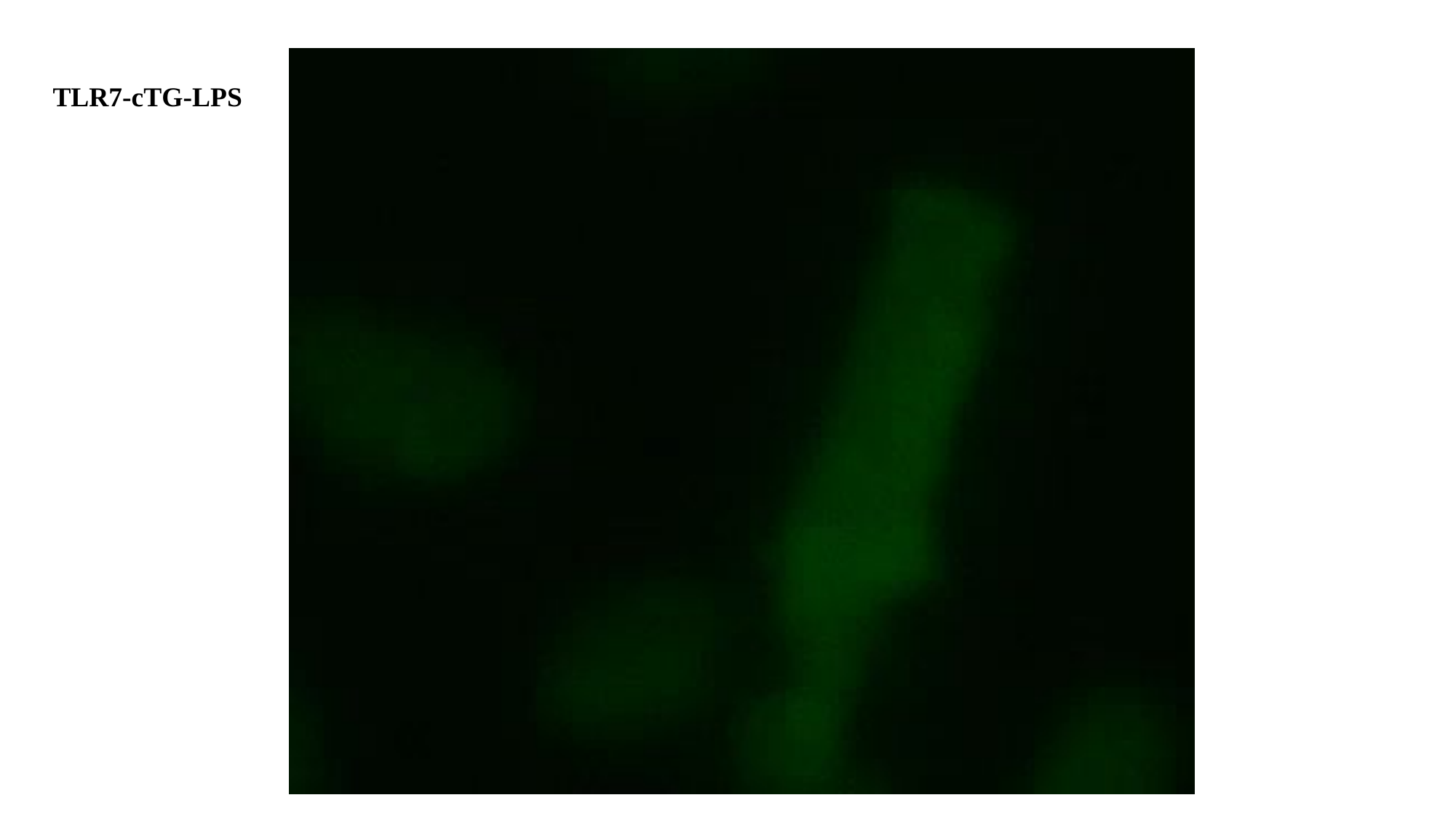

TLR7-cTG-LPS

## Slide 5
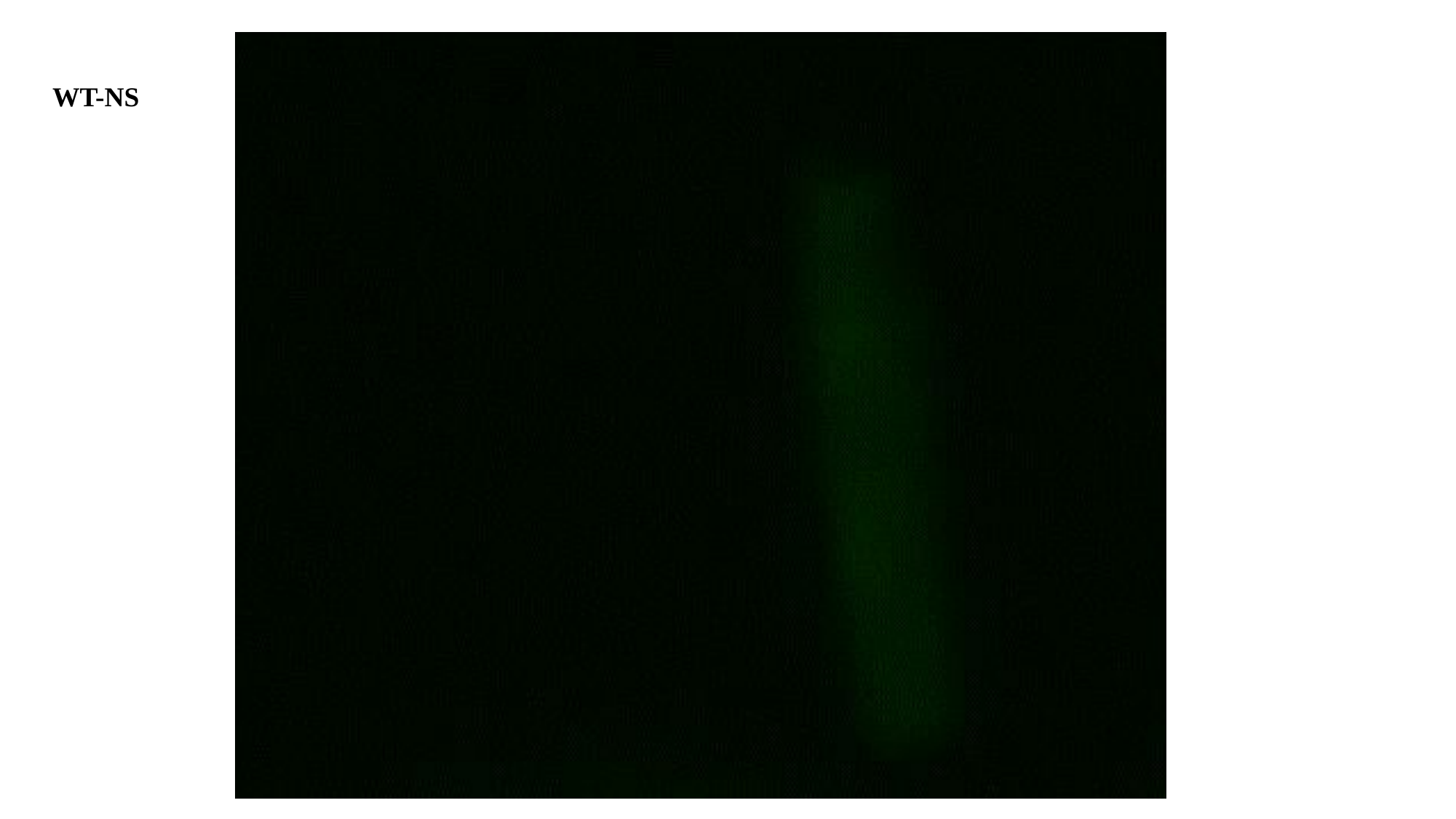

WT-NS

## Slide 6
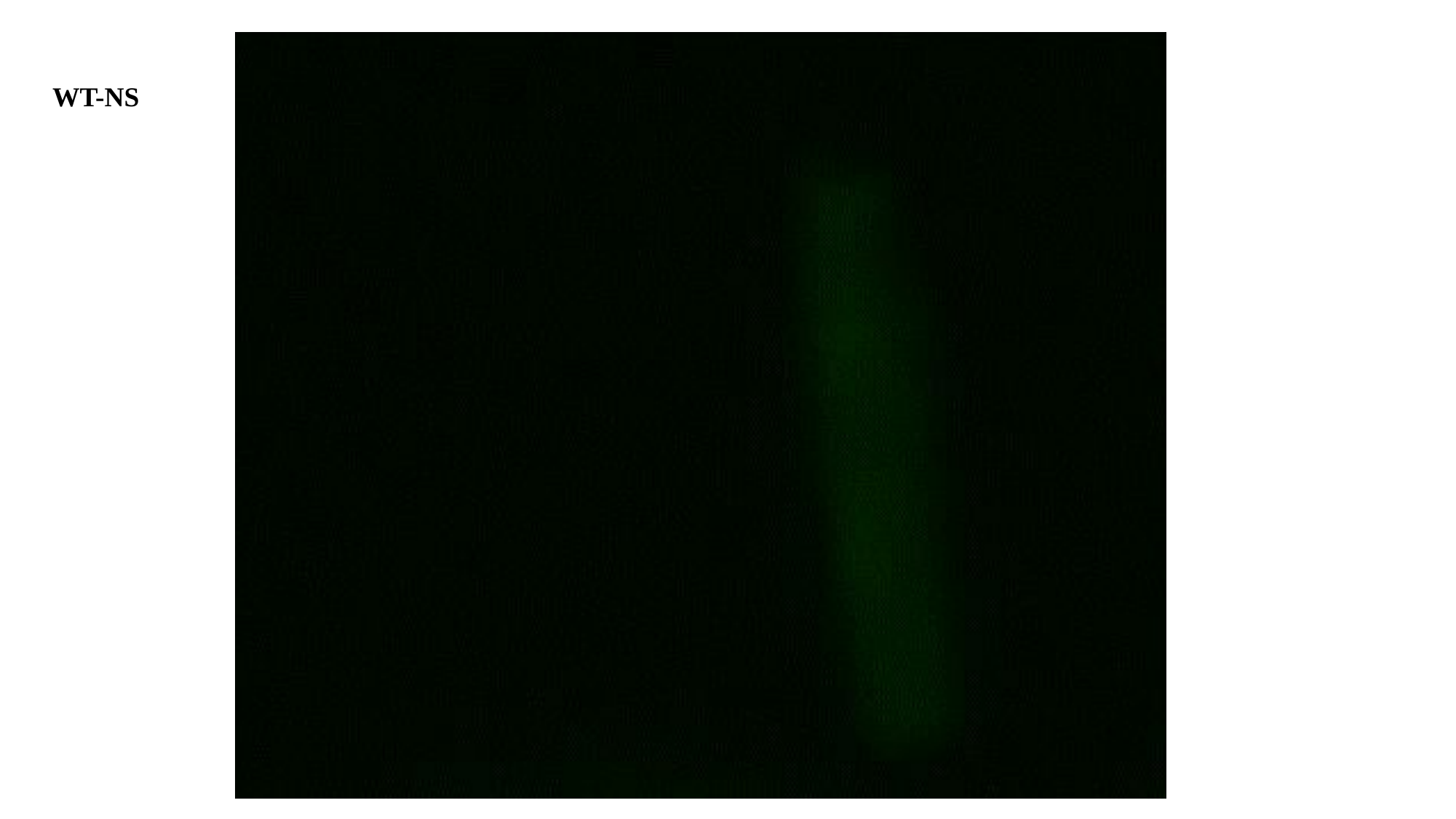

WT-NS

## Slide 7
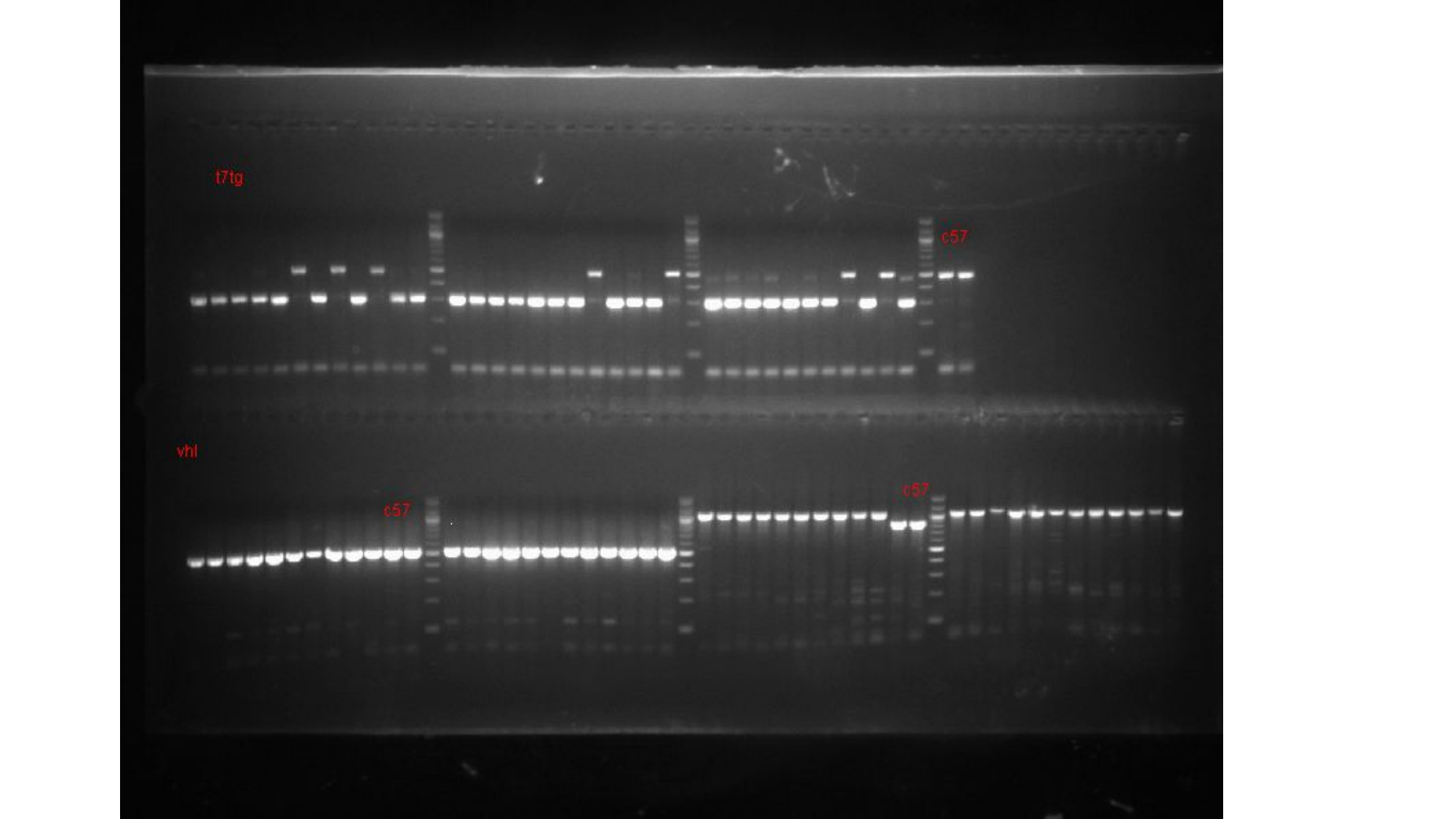

## Slide 8
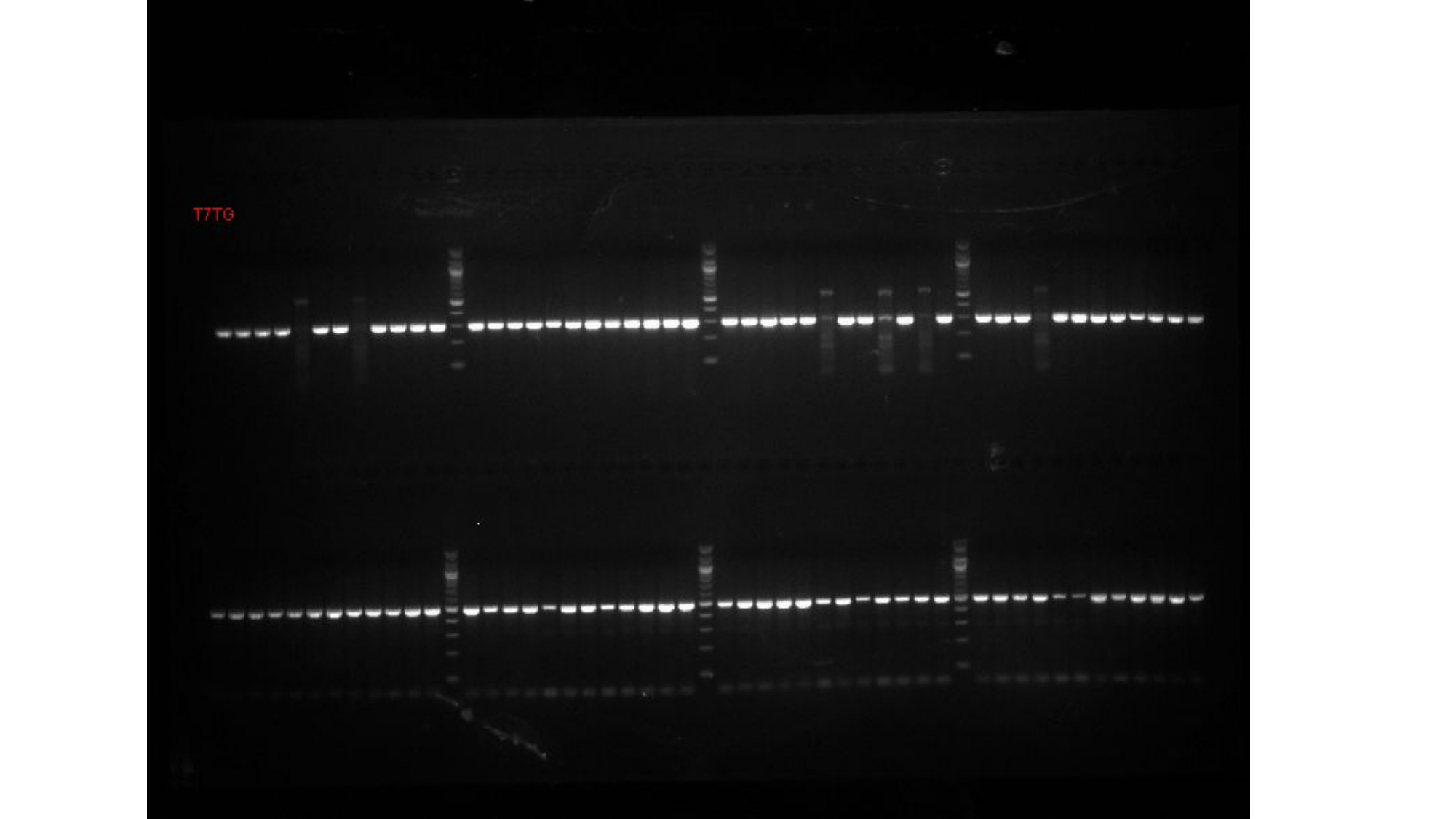

## Slide 9
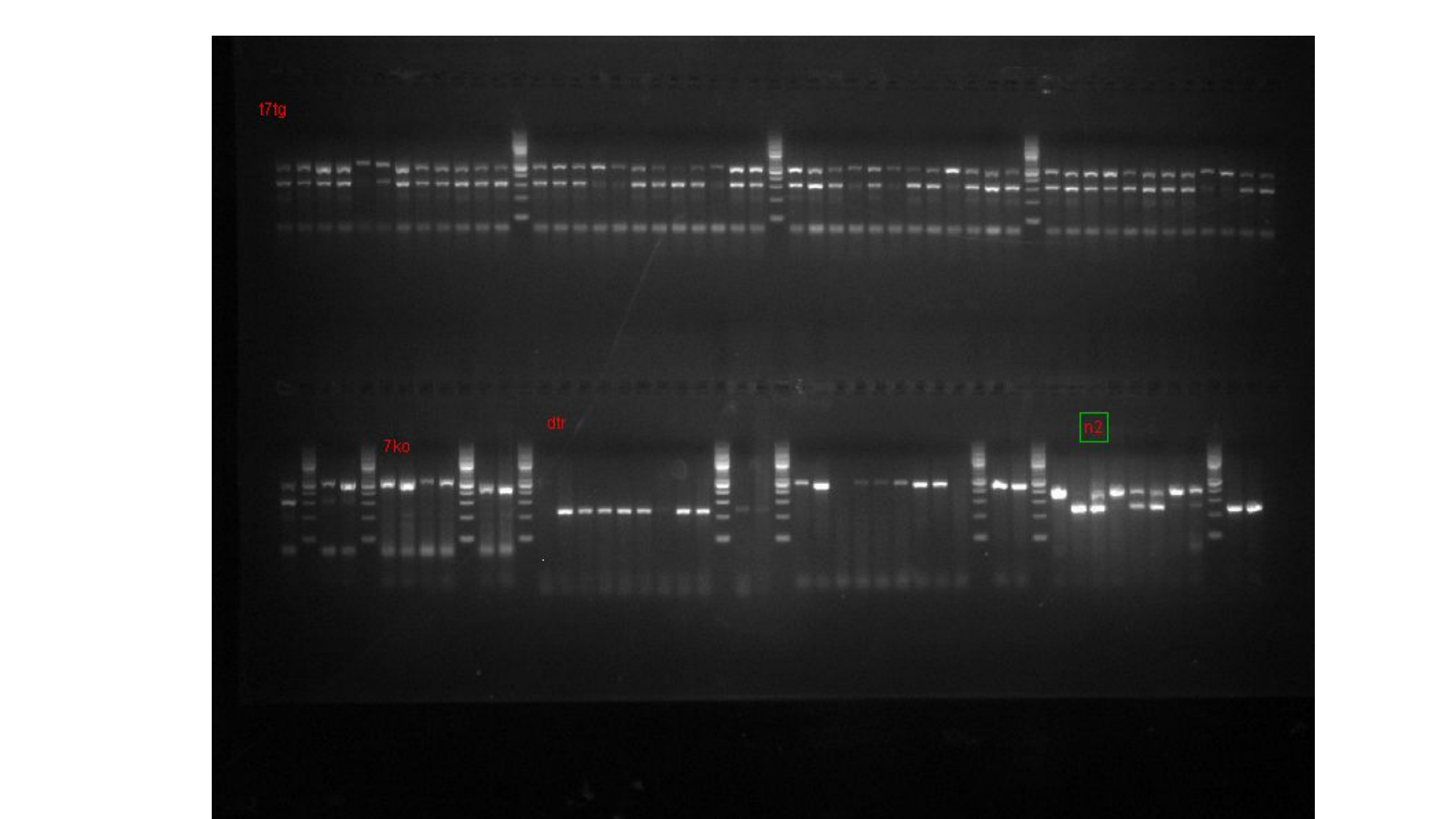

## Slide 10
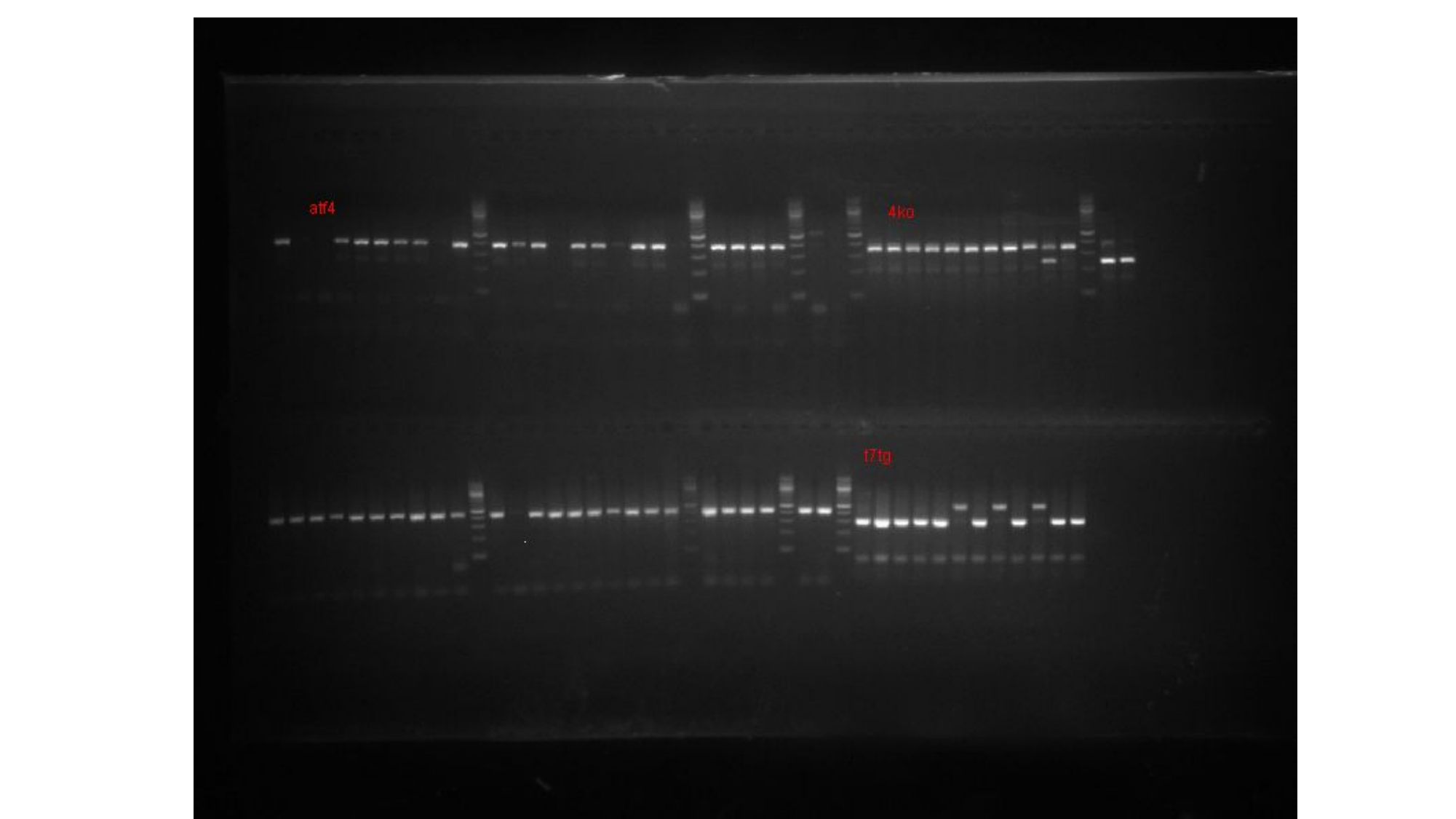

Supplement: Supplementary file 1 — Supporting Inforamtion [file CTM2-11-e266-s001.pptx]
