# Supplementary material for: Activation of Toll‐like receptor 7 provides cardioprotection in septic cardiomyopathy‐induced systolic dysfunction
Source: Clin Transl Med. 2021 Jan 1;11(1):e266. doi: 10.1002/ctm2.266 (PMC7775988; doi:10.1002/ctm2.266)
Supplement: Supplementary file 2 — Supporting Inforamtion [file CTM2-11-e266-s002.docx]

**Activation of Toll-like Receptor 7 Provides** **Cardioprotection in Septic Cardiomyopathy-induced Systolic Dysfunction**

**Running title:** Role of TLR7 in Septic cardiomyopathy

Xie et al.

1. Department of Cardiology, Renmin Hospital of Wuhan University, Wuhan 430060, RP China

2. Hubei Key Laboratory of Metabolic and Chronic Diseases, Wuhan 430060, RP China

3. Department of Cardiology, The Fifth Affiliated Hospital of Xinjiang Medical University, Ürümqi, China

4. Key Laboratory of Cardiovascular Remodeling and Function Research, Chinese Ministry of Education and Chinese Ministry of Health, Qilu Hospital of Shandong University, Jinan, 250012, China.

5. Department of Critical Care Medicine, Qilu Hospital of Shandong University, 107 Wenhuaxi Road, Jinan, 250012, People's Republic of China.

*These authors contributed equally to this work.

#Corresponding author: Deng Wei Email: [vivideng1982@whu.edu.cn](mailto:vivideng1982@whu.edu.cn) and Tang Qizhu Email: [qztang@whu.edu.cn](mailto:qztang@whu.edu.cn).

Address: Department of Cardiology, Renmin Hospital of Wuhan University, Jiefang Road 238, Wuhan 430060, P.R. of China. Tel.: +86 2788073385; Fax: +86 2788042292.

*Requests by researchers to access the data, analytic methods, and study materials for the purposes of reproducing the results or replicating procedures can be made to the*

*corresponding author who manages the information.*

**This PDF file includes:**

- **Supplementary Methods**
- **Supplementary Tables:**

**Supplementary Table 1.** Primary antibodies used in this study.

**Supplementary Table 2.** Primers used in qPCR.

**Supplementary Table 3.** Echocardiographic and hemodynamic parameters in WT and TLR7^-/-^ mice after LPS injection for 24h.

**Supplementary Table 4.** Echocardiographic and hemodynamic parameters in LWT and TLR7-cTG mice after LPS injection for 24h.

- **Supplementary Figures and legend**

**Supplementary Figure 1.** TLR7 expression was up-regulation *in vivo and in vitro*.

**Supplementary Figure 2.** The effects of TLR7 deficiency on inflammation, apoptosis and oxidative stress *in vivo*.

**Supplementary Figure 3.** TLR7 knockout regulated Ca2+ handling in AMCMs.

**Supplementary Figure 4.** TLR7 knockout controlled cAMP/PKA/PLN pathway.

**Supplementary Figure 5.** TLR7 knockout regulated Ca2+ handling in NRCMs.

**Supplementary Figure 6.** Cardiac TLR7 overexpression regulated cAMP/PKA/PLN pathway and Ca2+ handling.

- **Supplementary References**

**Supplementary Methods**

***Animals***

8-10 weeks old male wild type (WT) C57BL/6J mice, purchased from the Institute of Laboratory Animal Science, Chinese Academy of Medical Sciences (Beijing, China), kept in quarantine room for a week to adapt to the environment. Furthermore, transgenic mice with cardiomyocyte-specific TLR7 overexpression (TLR7-cTG) and TLR7 knockout (TLR7^−/−^) mice were utilized as well. A TLR7 cardiac-specific expression vector PBSIISK-aMHC-TLR7 was constructed to establish a TLR7 cardiac -specific transgenic mouse. METHODS: The TLR7 gene CDS of mice was cloned by qPCR, and the TLR7 gene was inserted into the downstream of the cardiac specific expression gene αMHC promoter to construct a cardiac -specific expression vector. The PBSIISK-aMHC-TLR7 was linearized with Notl, and the linearized cardiac tissue-specific expression vector was microinjected into the pronucleus of the mouse fertilized egg by microinjection. The genotype of the first transgenic mouse was detected by qPCR, and the specific expression of the murine TLR7 gene in heart of transgenic mice was verified by western blot. After identification of nucleic acids, Littermates wild type (LWT) mice were used as study controls. The detailed information was provided in attachment.

***Antibodies and reagents***

Primary antibodies used in this study include as follow: Toll-like receptor 7[ (TLR7), Thermo Fisher Scientific, PA5-95258], GAPDH (CST, 2118), Serca (Abcam, ab2816), Phospholamban[pSer16-PLN(phosphorylate Serine16), Abcam, ab15000], Phospholamban[pThr17-PLN(phosphorylate Threonine17), Abcam, ab62170], Phospholamban[PLN (Abcam, ab2865), Ryanodine Receptor 2 (RyR2,Abcam, ab2868), TLR7 (Abcam,ab124928),β-actin (proteintech, 600008-1-lg), TLR7 (proteintech, 17232-1-AP), Ca^2+^/calmodulin-dependent protein kinases [CaMKII (Abcam, ab52476)], Inducible nitric oxides synthase[iNOS (Abcam, ab15323)], HMGB1 (Abcam,ab79823), CD68 (Abcam, ab125212). PKA inhibitor H89 (MCE, HY-15979). Loxoribine (MCE, HY-108472). The secondary antibody used for western blot was purchased from LI-COR Biosciences, whereas anti-rabbit/mouse EnVisionTM+/HRP reagent used for immunohistochemistry was obtained from Gene Technology (Shanghai, China).

***TLR7 agonist (Loxoribine) and antagonist (IRS661) intervention***

TLR7 agonist (Loxoribine) and antagonist (IRS661) were utilized to explore the effect of TLR7 as previously described [1]. Briefly, AMCMs isolated from WT and TLR7-/- mice were incubated with TLR7 agonist (Loxoribine) 20 μg/ml or DMSO, and then the cAMP activity was assessed cAMP activity assay Kit. Additionally, AMCMs isolated from WT mice were subjected to LPS (10μg/ml) or PBS, Subsequently the AMCMs were incubated with TLR7 antagonist IRS661(9μg/ml): 5’-TGCTTGCAAGCTTGCAAGCA-3’, and the control: 5’-TCCTGCAGGTTAAGT-3’ (RiboBio, Guangzhou, China). To verified the efficiency, TLR7 protein level was detected by WB after loxoribine or IRS661 administration.

***DNA agarose gel electrophoresis***

DNA fragmentation caused by cell damage in NRVMs was assessed by DNA agarose gel electrophoresis as previously described [2]. Briefly, cell pellet was harvested and add with cell lysate, then centrifuge and collect supernatant, incubating with 1% SDS and RNaseA (5mg/ml) 56°C for 2h and proteinase K (2.5mg/ml) 37°C for 2h. Subsequently, sodium acetate and cold absolute ethanol were used to precipitate DNA overnight at 4°C. And eventually, DNA precipitation was dissolved with Tris+EDTA buffer and DNA loading buffer. Next, DNA sample was detected by 1.2% agarose gel electrophoresis and Ethidium bromide (EB) staining.

***Ca^2+^ transients and SR Ca^2+^ content***

Cardiomyocytes were loaded with 10μM Fluo-4 AM (Invitrogen) and imaged by a Leica AF6000 fluorescence microscope. Briefly, Cardiomyocytes were recalcified (0.8 mmol/L), then a total of 30 ml of cell liquid and the impurities were filtered through a sieve stored in a 50 ml centrifuge tube or a beaker at room temperature. Then the cell liquid was divided into two 15 ml centrifuge tubes, and centrifuged at 1000-1200 rpm for 3-5 min in a centrifuge, and discarded the supernatant and resuspended with 0.8 mmol/L bench solution, and then transferred in 1 ml to 1.5 ml EP tube. And we took 8-10ul of Fluo-4, AM mother liquoring to 1.5ml EP tube containing 1ml cardiomyocyte solution (Fluo-4, AM concentration is 10umol/L), and incubated in a 35-37 °C water bath for 30-60 min, and mixed and inverted once during the interval of 10 min so that the cells and probes were in full contact. For the staining agent, Each 1.5 ml EP tube was added with 1 ml of normal bench-top solution containing 0.2 mM anion inhibitor sulfinpyrazone, and 0.25 ml of Fluo-4 AM-incubated cardiomyocyte solution was added to the first EP tubes. Then the solution was centrifugated at 4 °C in 800-1000 rpm for 3 min. And then discarded the supernatant, 1 ml of a normal benchtop solution containing 0.2 mM anion inhibitor sulfinpyrazone was added to each of the four EP tubes, and the mixture was mixed by blowing and centrifuged again. And then added 1ml of normal Tyrode solution in the well, and aspirated 0.1ml of cell suspension so that made it stand for 2-3min until the cells were attached, and selected the cells under the microscope (electrical stimulation 1-2 times to observe its contraction, horizontal Good pattern detection, and marked with aperture on the computer display). And the stimulation voltage was 20V, and performed with 1.0Hz. For the detection of SR Ca^2+^ load/content: 10x caffeine solution to 100ul stimulation.

***Ca^2+^uptake measurement***

Ca^2+^ uptake was measured by a Fura-2 based as previously described [3]. Briefly, microsomes were incubated in assay buffer [100 mM KCl, 10 mM HEPES-KOH (pH 7.4), 10 mM oxalate, 5 mM MgCl_2_, 10 μM ruthenium red and 2 μM Fura-2 free acid (Beyotime Biotechnology, China)]. The uptake reaction was initiated by addition of 5 mM ATP-Na^+^ (Sigma) and 2 μM Ca^2+^. The fluorescence ratio (excitation at 340 and 380 nM) was recorded at 510 nM emission using a fluorescence microplate reader (Synergy HT, Bio-tek, Winooski, VT, United States). The rate of Ca^2+^ uptake into microsomes was calculated by measuring the linear portion of the slope after addition of Ca^2+^ as previously described [4].

***Cardiomyocyte Sarcomere Shortening***

Sarcomere shortening was recorded simultaneously on an IonOptix system (Milton, MA). Adult cardiomyocytes were incubated with membrane permeable fluorescent indicator fura-2 AM (2 μM) and probenecid (0.5 mM). Cardiomyocytes were perfused with 1.2 mM Ca2+ Tyrode solution and electrically paced at 1, 2, 4, and 6 Hz via platinum wires. The sarcomere shortening were analyzed based on single-cell-averaged tracing. The final values were derived from 10 to 15 individual cells in each group and calculated for statistical analysis. At least three mice from each mouse group were used to prepare cardiomyocytes for the functional studies.

***Microsome isolation and Serca2a-ATPase activity assay***

Microsomes containing crude ER membrane vesicles were isolated as previously described[5, 6]. The ATPase activity of Serca2a was determined via measurement of inorganic phosphate (Pi) resulted from ATP hydrolysis as previously described43. Briefly, prepared 1 mL of buffer I (Tris-HCl 30 mmol/L, sucrose 0.3 mol/L, leupeptin 5 mg/L, PMSF 0.1 mmol/L, pH 7.0) added into 200 mg of myocardial tissue. It was ground to homogenate at 4 °C, centrifuged at 5500 g for 10 min, and the supernatant was centrifuged at 4°C for 20 min. The supernatant was discarded, and the precipitate was taken. 1 mL of buffer II (Tris-HCl 30 mmol) was added as well. The composition of Buffer II involves sucrose 0.3 mol / L, leupeptin 5 mg / L, PMSF 0.1 mmol / L, KCL 0.6 mol / L, pH 7.0), fully mixed at 4 ° C, 143000g centrifugation for 45 min; discarded Clear the supernatant, collect the precipitate, add 1 mL of buffer III (Tris-maleic acid 20 mmol/L, sucrose 0.3 mol/L, leupeptin 5 mg/L, PMSF 0.1 mmol/L, KCl 0.1 mol/L pH 7.0 ), fully mixed to form sarcoplasmic reticulum vesicles. Then we utilized the G6P quantification kit (Beyotime Biotechnology, China) to detect the activity of SR-labeled enzyme G6P extracted by sucrose differential centrifugation, determine the purity of SR, and have been prepared by BCA protein concentration method. The sarcoplasmic reticulum, using the inorganic phosphate method (ultra-micro ATPase activity kit, Nanjing Jiancheng Institute of Bioengineering) to determine the activity of the sarcoplasmic reticulum calcium pump, strictly according to the kit instructions Operation. The reagents, water bath, centrifugation, and standing were sequentially added. Finally, the absorbance values ​​of the tubes were measured by a fluorescence microplate reader (Synergy HT, Bio-tek, Winooski, VT, United States) at 636 nm and a light path of 0.5cm.

***Adenovirus infection and siRNA transfection***

Adenoviral vector loaded with TLR7 (Ad-TLR7) was packaged from Vigene Biosciences (Jinan, China). NRVMs and adult cardiomyocytes were infected with adenovirus particles at MOI of 50 and Ad-LacZ was used as a control, as previously descripted [7]. Briefly, after resuspended the cells in the logarithmic growth phase, the cells were inoculated in a 12-well plate at a density of 1*10^5^/L, and kept it growth overnight, then removed 70-80% of the culture solution in the 12-well plate to replace the fresh medium. At the same time, the virus solution diluted with PBS concentration gradient was added, and evenly mixed to place in an incubator culture. Total RNA was extracted when the cells were incubated for 24h, and virus infection efficiency was detected by qPCR. Small siRNA oligos (RiboBio, Guangzhou, China) were transfected with Lip6000 reagent (Beyotime Biotechnology, China) following manufacturer’s protocol. Briefly, 1~5*10^5^ cells were inoculated into a 6-well plate or 0.5~1*10^4^ cells into a 24-well plate containing appropriate amount of complete medium to achieve a cell density of 30% to 50% during transfection. After the siRNA was diluted, we added lip6000-DMEM for 5 min at room temperature, then added the mixed liquid into each well, and changed medium after transfected for 6h, and continued to be cultured for 48-72 hours, as previous study described [8]. QPCR was used to detect the transfection efficiency of siRNA as well.

***Measurement of intracellular cAMP activity***

To measure intracellular [cAMP], *S. coelicolor* A3(2) was grown in R2YE liquid media for 48 h at 28 ℃ and sub cultured into fresh R2YE liquid media (1% final volume of inoculation). A 1-mL sample from each culture was harvested every 24 h at 4 ℃ by centrifugation for 2 min at 6000 g Samples were then washed with PBS buffer (0.8% NaCl, 0.02% KCl, 0.15% Na2HPO4, and 0.024% KH2PO4 at pH 7.4) and resuspended in 1mL ATP-free water. cAMP activity was measured using the cAMP-GloTM assay (Promega) and a GLOMAXTM 96 microplate luminometer (Promega) according to the manufacturer’s instructions, with the following exception: an additional 10 mL of 50mg/mL lysozyme was added to the lysis buffer and further incubation was performed for 15 min at 37 ℃. DCW was measured and values are expressed as intracellular [cAMP] g^-1^ DCW.

***Western blot and Immunoprecipitation***

Isolated cardiomyocytes and myocardial tissue in indicated groups were lysed in radioimmunoprecipitation assay (RIPA) buffer (Servicebio, China). The composition of the RIPA buffer (in mmol/L) was: Tris (20), NaCl (150), EDTA (5), EGTA (5), DTT (1) plus 1.0% Triton X-100 and 0.5% deoxycholate. Myocardial tissue was disrupted in extraction buffer (0.1 M Tris–HCl, 0.01 M EDTA, 0.04 M DTT, 10% SDS, pH 8.0) by an ultrasonic device. The composition of protease inhibitors (in mmol/L) were: Na^+^ pyrophosphate (2.5), β-glycerophosphate (1.0), Na3VO4 (1.0), PMSF (1.0), NaF (2.0), and 10μg leupeptin. one tablet of a protease inhibitor cocktail (Complete Mini, Roche, Germany) and one tablet of phosphatase inhibitor (Phos-stop, Roche, Germany) were added into the mixture with per 10 mL buffer. The buffer was adjusted to pH 7.4. Lysates to assay for protein concentration by the bicinchoninic acid assay. BSA was used to produce a standard curve. In each group, 20μg protein lysate was prepared for SDS-PAGE, and then the proteins were electrophoresed and transferred to PVDF membrane (Millipore, USA). After blocked for an hour, the primary antibody was incubated at 4°C overnight. Next day, the chemical secondary antibody was incubated at room temperature for an hour, and the chemiluminescent protein detection developer (ECL, Broad, USA) was utilized with a chemiluminescence scanning membrane apparatus to obtain protein bands. Image J software was used to analysis western blot strips. For immunoprecipitation, 20~30mg myocardial tissue was cut into pieces, and then added with 1.0mL IP lysate, and lysed for 30min at 4°C, and collected lysate with 1μg mouse IgG and 20μl fully resuspended protein A+G Agarose (Beyotime Biotechnology, China), and shake slowly at 4°C for 30min, then the lyse was centrifuged for 5 min in 2500rpm and then collected the supernatant, and added with 1:800 dilution of the primary antibody to a 4 °C shaker overnight, and 40μL of lyse was resuspended with Protein A+G Agarose in next day, and slowly shake at 1 °C for 1 hour at 2500 rpm centrifuge for about 5 minutes, and carefully aspirated the supernatant, then washed the pellet 5 times with the lysate or PBS by preparing the protein sample. Then removed the supernatant, and added 20-40 μL of 1x SDS-PAGE electrophoresis loading buffer Vortex to resuspend the protein. When the protein was precipitated, the sample was centrifuged to the bottom of the tube by instantaneous high-speed centrifugation. After treated at 100°C or boiling water bath for 3-5 minutes, some or all of the samples were taken for SDS-PAGE electrophoresis as mentioned above.

***RNA purification and qPCR***

Total RNA was purified from tissue or cultured cell with Trizol (Invitrogen) following manufacturer’s protocol. A part of total RNA was used for reverse transcription with SSTIII kit (Invitrogen) to generate cDNA. The cDNA is used in SYBR-based real-time qPCR on the following cycling conditions: hold for 2 min at 50°C followed by 10 min at 95°C to heat-start the *Taq* polymerase enzyme, then 40 cycles of 95°C for 10 min, 60°C for 1 min. Relative mRNA levels were analyzed using comparative Ct calculations normalized to GAPDH or β-Actin. The sequences of the primers for each gene detected are listed in Supplementary Table 1.

***Statistical analysis***

All data in this study are presented as mean ± standard error of the mean (SEM) and were evaluated by linear mixed modeling (IBM SPSS Statistics, version 22). One-way analysis of variance (ANOVA) followed by Tukey post hoc test was used when comparing multiple groups, and differences between two groups were evaluated by unpaired Student’s t-test. A P-value < 0.05 was considered statistically significant.

**Supplementary Tables:**

**Supplementary Table 1.** Primary antibodies used in this study

**Table S1. Primary antibodies used in this study**

| **Antibody** | **Customer** | **Product number** | **Dilution** | **Application** |
| --- | --- | --- | --- | --- |
| TLR7 | Abcam | ab124928 | 1:500 | WB |
| TLR7 | Proteintech | 17232-1-AP | 1:500  1:200 | WB  IF |
| TLR7 | Invitrogen | PA5-95258 | 1:200 | IF |
| β-actin | Proteintech | 600008-1-1g | 1:1000 | WB |
| GAPDH | Cell Signaling Technology | 2118 | 1:1000 | WB |
| RyR2 | Abcam | Ab2868 | 1:1000  1:250 | WB  IF |
| Serca | Abcam | Ab2816 | 1:1000  1:250 | WB  IF |
| pSer16-PLN | Abcam | Ab15000 | 1:1000  1:250 | WB  IF |
| pThr17-PLN | Abcam | Ab62170 | 1:1000 | WB |
| PLN | Abcam | Ab2865 | 1:1000  1:200 | WB  IF |
| CaMKII | Abcam | Ab52476 | 1:1000 | WB |
| iNOS | Abcam | Ab15323 | 1:1000 | WB |
| HMGB1 | Abcam | Ab79823 | 1:1000 | WB |
| CD68 | Abcam | Ab125212 | 1:200 | IF |
| CD4 | Abcam | Ab25617 | 1:200 | IF |
| C-caspase-3 | CST | 9661 | 1:1000 | WB |
| NOX2 | Abcam | Ab129068 | 1:1000 | WB |
| Bax | CST | 2772 | 1:1000 | WB |
| Caspase-3 | CST | 9662P | 1:1000 | WB |
| TLR4 | SANTA | Sc-30002 | 1:500 | WB |

**Supplementary Table 2.** Primers and probes used in quantitative RT-PCR

**Table S2. Primers Used in qPCR**

| Gene | Species | Forward primer (5‘→3') | Reverse primer (5‘→3') |
| --- | --- | --- | --- |
| TLR7  TLR7 | Rat  Mouse | AGGCTTCAGTGGTTTGGATG  AGGGCCTCTTCTGCGATTTC | CACCAACAGCACCATCGTTA  CTTTGGAAGGACTCACCGCT |
| PKA  PKA | Mouse  Rat | GCACAGTACGTCAGGAAAGC  GGAGCCTAATTAGCAAGCCAC | AAGCCGAGCATTCCATCTGT  CGGAAACCCATAAGAAGGGC |
| PKC | Mouse | CTACGACGATATGGCCTCCG | GGTAGCGGTAGTAATCGCCC |
| ATP2A2 | Mouse | ACTTCTTGATCCTCTACGTG | AAATGGTTTAGGAAGCGGTT |
| ATP2A2  RyR2  RyR2  TNFα  IL-1β  IL-6  MCP-1  GAPDH  GAPDH | Rat  Mouse  Rat  Mouse  Mouse  Mouse  Mouse  Mouse  Rat | GGAGGACAACCCAGACTTCG  CCTTCCTGGCATAATGGAAA  GTAGCCCACAAAGGAGTCCC  GACATGCCGCCTGGAGAAAC  TGGTACATCAGCCCGAAC  GTCAGCTGGATAGCGACA  TGGCTCAGCCAGATGCAGT  ACTCCACTCACGGCAAATTC  GACATGCCGCCTGGAGAAAC | AGCACAAAGGGCCAGGAAAT  CATGTTGCAGGTCTGGAGTG  CTTCTCAACCTGTGCGTTGC  AGCCCAGGATGCCCTTTAGT  GTCAGCTGGATAGCGACA  GAAGCACAGGAGCAGGTGTAGA  CCAGCCTACTCATTGGGATCA  TCTCCATGGTGGTGAAGACA  AGCCCAGGATGCCCTTTAGT |

**Supplementary Table 3.** Physiological and echocardiographic parameters.

**Table S3.** **Echocardiographic and Hemodynamic parameters after LPS injection 24h**

|  | WT-NS  (n=9)  Mean±SEM | TLR7^-/-^-NS  (n=9)  Mean±SEM | WT-LPS  (n=8)  Mean±SEM | TLR7^-/-^-LPS  (n=8)  Mean±SEM |
| --- | --- | --- | --- | --- |
| **Echocardiographic parameter** | | | | |
| LVIDd, mm | 3.85±0.08 | 3.78±0.07 | 4.75±0.07* | 4.87±0.09^#^ |
| LVIDs, mm | 2.25±0.07 | 2.31±0.09 | 3.01±0.08* | 3.51±0.09^#^ |
| IVSs, mm | 0.94±0.11 | 0.96±0.09 | 1.23±0.15* | 1.04±0.12 |
| LVPWd (mm) | 0.67±0.06 | 0.65±0.09 | 0.68±0.08 | 0.66±0.08 |
| LVPWs (mm) | 0.94±0.11 | 0.96±0.07 | 0.81±0.10* | 0.73±0.11^#^ |
| EF (%) | 85±1 | 84±1 | 54±2* | 47±2^#^ |
| FS (%) | 44±1 | 45±2 | 27±2* | 21±2^#^ |
| **Hemodynamic parameter** | | | | |
| HR, bpm | 584±21 | 579±23 | 542±18* | 547±25 |
| CO (μL) | 10829±74 | 9728±68 | 6034±95* | 4573±83^#^ |
| ESV | 9.5±0.52 | 10.2±0.74 | 18.2±0.93* | 28.2±0.79^#^ |
| EDV | 14.8±0.37 | 13.1±0.26 | 24.7±0.78* | 34.5±0.83^#^ |

Note: Mean ± SEM. WT-LPS VS WT-NS: *P<0.05. TLR7^-/-^-LPS VS WT-LPS months: ^#^P<0.05.

Abbreviations: HR, heart rate; LVIDd, left ventricular end-diastolic diameter; LVIDs, left ventricular end-systolic diameter; IVSs, interventricular septal thickness at end-systole; LVPWd, left ventricular posterior wall thickness at end-diastole; LVPWs, left ventricular posterior wall thickness at end systole; FS, fractional shortening; EF, ejection fraction. CO, cardiac output; ESV, end-systolic volume; EDV, end-diastolic volume.

**Supplementary Table 4.** Echocardiographic parameters

**Table S4.** **Echocardiographic parameters after 20 months in HSP47 overexpression.**

|  | LWT-NS  (n=12)  Mean±SEM | TLR7-cTG-NS  (n=12)  Mean±SEM | LWT-LPS  (n=12)  Mean±SEM | TLR7-cTG-LPS  (n=12)  Mean±SEM |
| --- | --- | --- | --- | --- |
| **Echocardiographic parameter** | | | | |
| LVIDd, mm | 3.79±0.09 | 3.81±0.11 | 4.73±0.12* | 4.57±0.10^#^ |
| LVIDs, mm | 2.27±0.08 | 2.29±0.12 | 3.07±0.09* | 2.56±0.11^#^ |
| IVSs, mm | 0.92±0.12 | 0.91±0.09 | 1.25±0.12* | 1.01±0.10 |
| LVPWd (mm) | 0.68±0.06 | 0.69±0.09 | 0.68±0.09 | 0.68±0.11 |
| LVPWs (mm) | 0.95±0.10 | 0.96±0.11 | 0.79±0.12* | 0.85±0.10^#^ |
| EF (%) | 82±1 | 82±1 | 60±2* | 69±2^#^ |
| FS (%) | 39±2 | 41±1 | 31±2* | 36±2^#^ |
| **Hemodynamic parameter** | | | | |
| HR, bpm | 571±20 | 568±24 | 541±17* | 543±23 |
| CO (μL) | 10153±47 | 10034±35 | 6128±73* | 8853±79^#^ |
| ESV | 9.9±0.37 | 10.6±0.64 | 26.7±0.81* | 21.6±0.86^#^ |
| EDV | 15.3±0.32 | 16.3±0.37 | 27.9±0.43* | 21.5±0.52^#^ |

Note: Mean ± SEM. LWT-LPS VS LWT-NS: *P<0.05. TLR7-cTG-LPS VS LWT-LPS months: ^#^P<0.05.

Abbreviations: HR, heart rate; LVIDd, left ventricular end-diastolic diameter; LVIDs, left ventricular end-systolic diameter; IVSs, interventricular septal thickness at end-systole; LVPWd, left ventricular posterior wall thickness at end-diastole; LVPWs, left ventricular posterior wall thickness at end systole; FS, fractional shortening; EF, ejection fraction. CO, cardiac output; ESV, end-systolic volume; EDV, end-diastolic volume.

**Supplementary Figures:**

**Suppl Figure 1.**


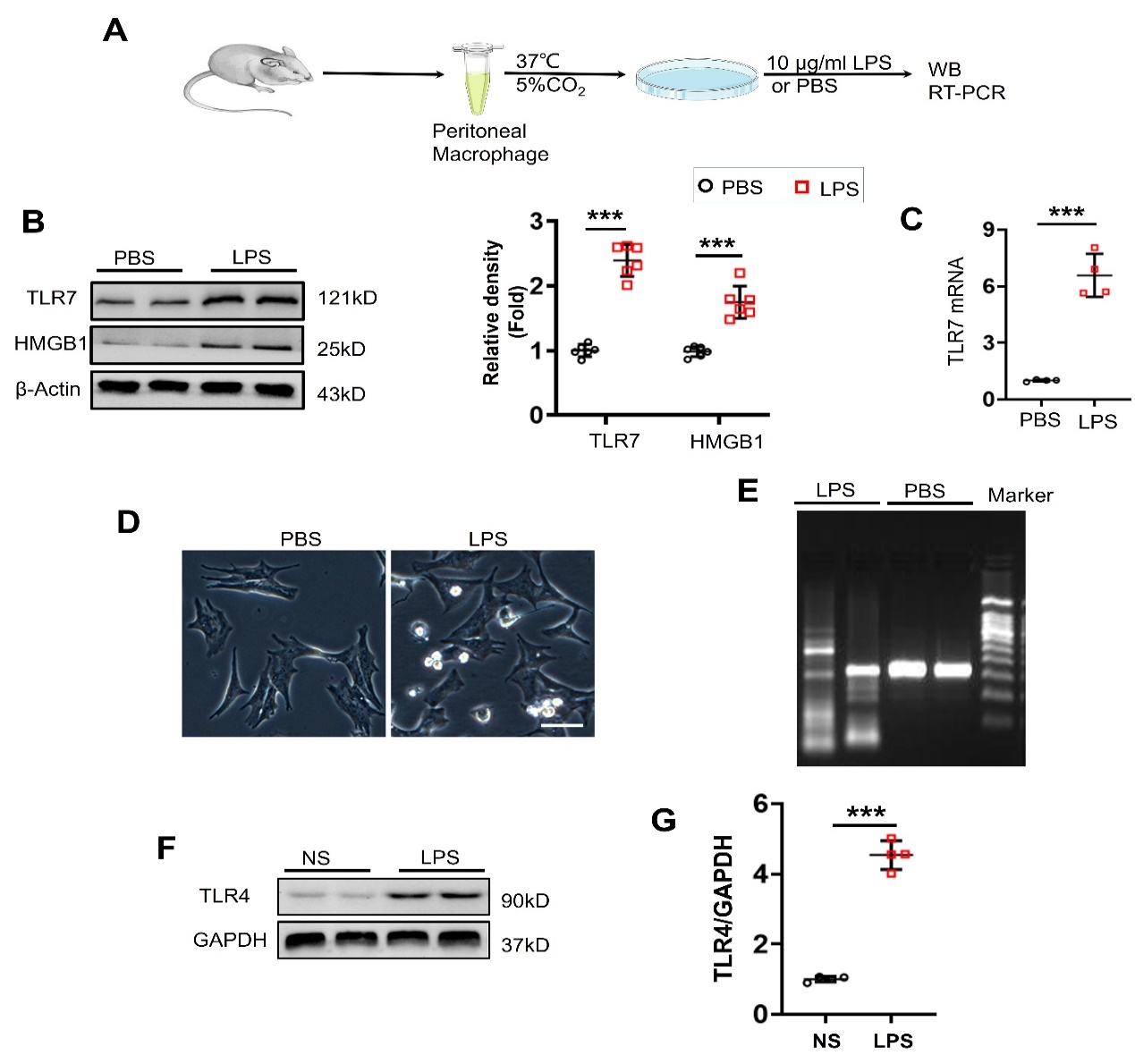


**Supplemental Figure 1:** Peritoneal macrophages isolated from C57/B6J mice were subjected to LPS or PBS. **(A)** Protocol. **(B)** Representative images of western blot and statistical analysis of TLR7 and HMGB1 expression in isolated peritoneal macrophages (n=6). **(C)** qPCR analysis of TLR7 mRNA level in isolated peritoneal macrophages (n=4). Normalized to β-Actin. **(D-E)** Neonatal rat ventricular myocytes (NRVMs) were stimulated with LPS (10 μg/mL) or PBS for 24 h. **(D)** Representative images of morphology of NRVMs. Scale bar: 20 μm. **(E)** Representative images of DNA agarose gel electrophoresis of NRVMs. **(F-G)** Representative images of western blot and statistical analysis of TLR4 level in myocardial tissue (n=4). The data are shown as the mean±SEM, with each data point representing a cell sample or a mouse. * indicates *p* < 0.05, ** *p* < 0.01, and *** *p* < 0.001.


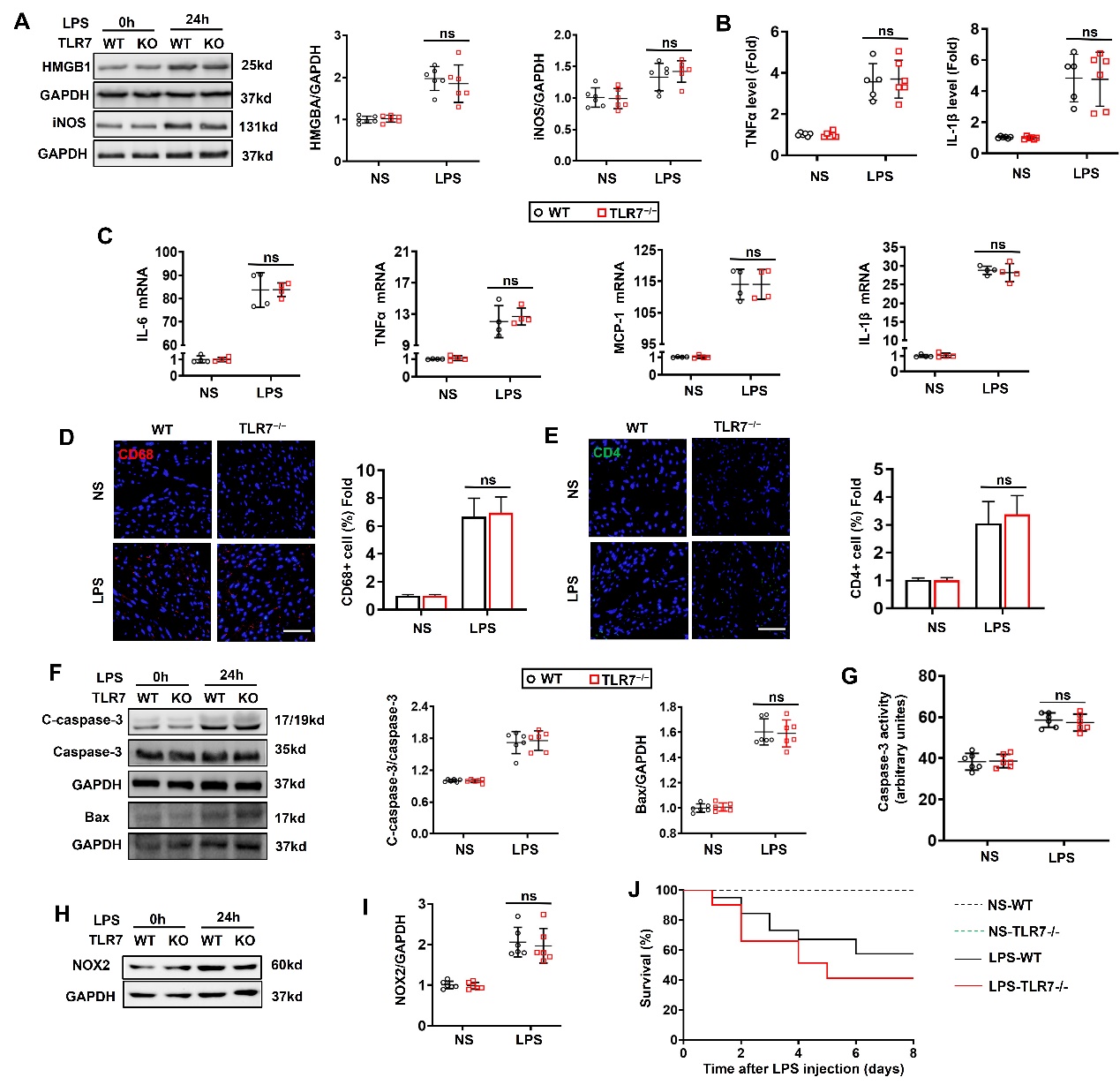


**Supplemental Figure 2:** C57/6BJ WT and TLR7^−/−^ mice were subjected to disposable intraperitoneal injection of LPS (10mg/kg) for 24h. **(A).** Representative images of western blot and statistical analysis of HMGB1 and iNOS expression (n=6). **(B)** Elisa analysis of TNFα and IL-1β in serum (n=6). **(C).** qPCR analysis of mRNA level of Interleukin-1β (IL-1β), IL-6, tumor necrosis factor α (TNFα) and monocyte chemoattractant protein-1 (MCP-1) (n=4). Normalized to GAPDH. (D-E) Representative images of immunofluorescence of CD68 and CD4 in myocardial tissue (n=18-26 from 3 mice) scale bar: 200 μm. **(F).** Representative images of western blot and statistical analysis of C-caspase-3, caspase-3 and bax expression (n=6). **(G).** Heart tissue lysates were harvested to detected caspase-3 activity. (n=6). (H-I). Representative images of western blot and statistical analysis of NOX2 and GAPDH expression (n=6). (J). Survival analysis post-LPS injection 8 days, with 12 mice in each group. P=0.0187, comparison of survival curves between LPS-WT and LPS-TLR7 KO mice using the log-rank (Mantel-Cox) test. The data show as the mean±SEM, with each data point representing a cell sample or a mouse. ns indicates no significance.


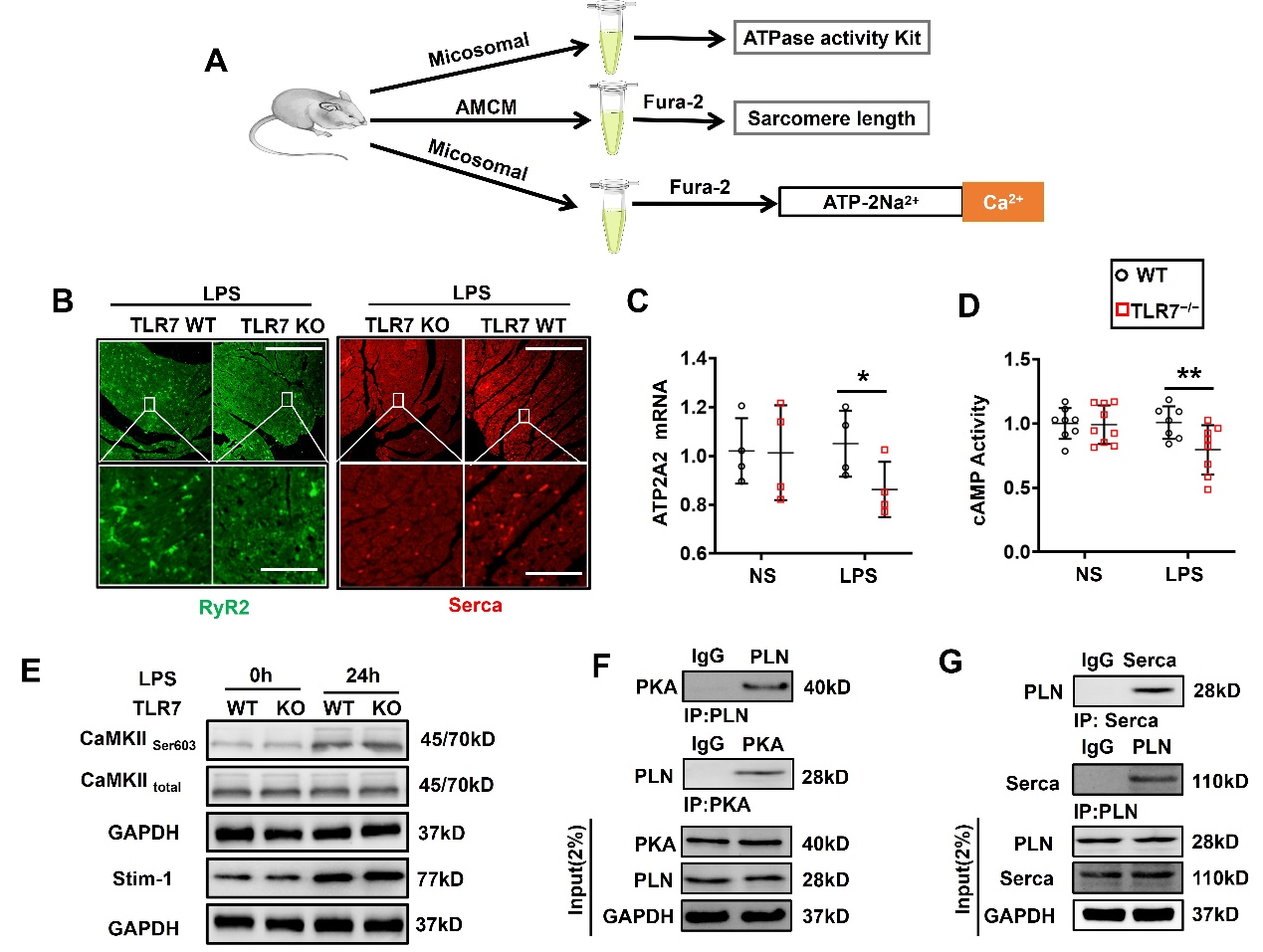


**Supplemental Figure 3:** After LPS stimulated for 24h, cardiac tissue and cardiomyocytes were harvested from C57BL/6J WT and TLR7^−/−^ mice. **(A).** Protocol. **(B).** Representative images of immunofluorescence of RyR2 and Serca (n=3-4). Upper scale bar: 200 μm and lower scale bar: 80 μm. **(C).** QPCR analysis of mRNA level for the gene coding for Serca (Atp2a2) (n=4). **(D).** cAMP activity was detected by cAMP-GloTM assay (Promega) and a GLOMAXTM 96 microplate luminometer (Promega) (n=6-10). **(E).** Representative images of western blot of phosphorylated serine16 calmodulin-dependent protein kinase II (CaMKII), total CaMKII and stim-1 (n=6). **(F).** PKA interacts with PLN in adult mice cardiomyocytes (ACMs). ACM lysates were immunoprecipitated with anti-PKA or anti-PLN antibodies and probed with anti-PKA or anti-PLN antibodies (n=6). **(G).** PLN interacts with Serca in ACMs. ACM lysates were immunoprecipitated with anti-PLN or anti-Serca antibodies and probed with anti-PLN or anti-Serca antibodies (n=6). The data show as the mean±SEM, with each data point representing a mouse. * indicates *p* < 0.05 and ** *p* < 0.01.


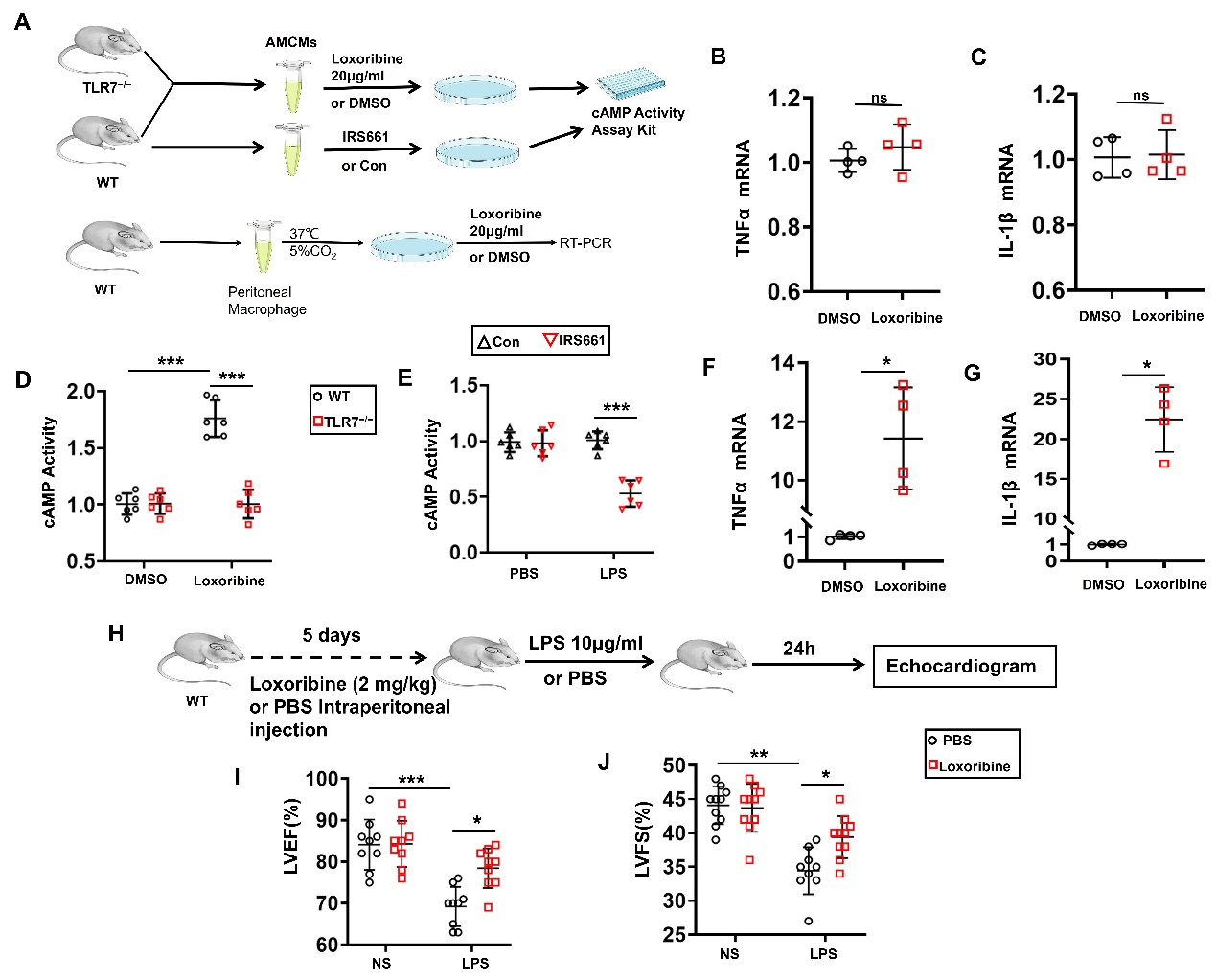


**Supplemental Figure 4:** AMCMs isolated from WT and TLR7-/- mice were incubated with TLR7 agonist (Loxoribine) or DMSO, and AMCMs isolated from WT mice were subjected to LPS or PBS before incubating with TLR7 antagonist IRS661. (A) protocol. **(B-C)** QPCR analysis of mRNA level for TNFα and IL-1β in isolated AMCMs after pretreated with Loxoribine (n=4). **(D)** cAMP activity was detected after loxoribine administration by cAMP-GloTM assay (Promega) and a GLOMAXTM 96 microplate luminometer (Promega) (n=6). **(E)** cAMP activity was detected after IRS661 administration by cAMP-GloTM assay (Promega) and a GLOMAXTM 96 microplate luminometer (Promega) (n=6). (F-G) QPCR analysis of mRNA level for TNFα and IL-1β in isolated peritoneal macrophages after pretreated with Loxoribine (n=4). (H-J) C57BL/6J mice were subjected to Loxoribine before LPS administration. (H) protocol. (I-J) Echocardiographic examination was used to evaluate cardiac function when LPS administrated for 24 h (n=8-12). **(I)** Left ventricular ejection fraction, **(J)** left ventricle fractional shortening (LVFS) were calculated from echocardiography. The data are shows as the mean ± SEM, with each data point representing a mouse. *** indicates *p* < 0.001.


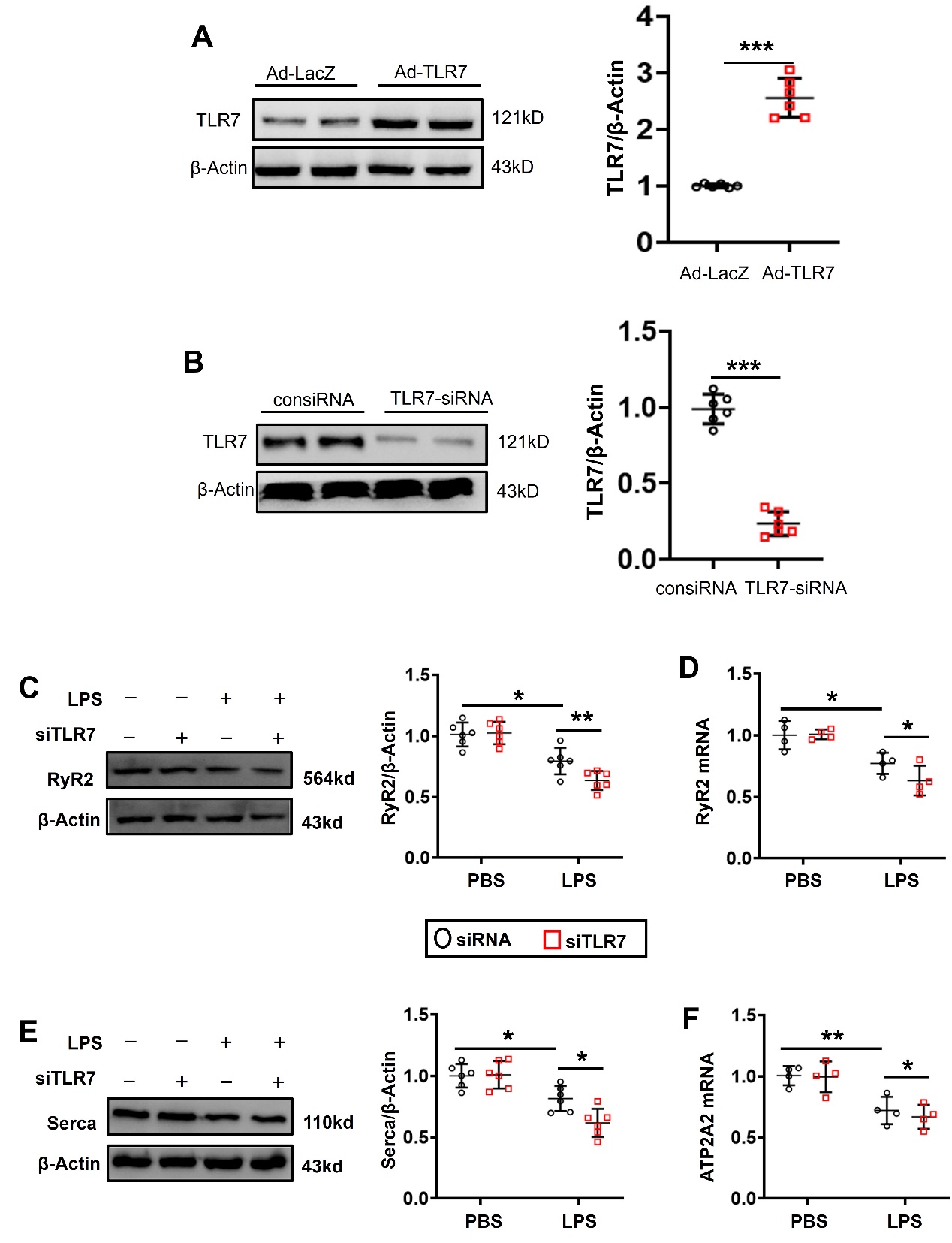


**Supplemental Figure 5: (A)** Representative images of western blot and statistical analysis of TLR7 expression after Ad-LacZ or Ad-TLR7 administration (n=6). **(B)** Representative images of western blot and statistical analysis of TLR7 expression after consiRNA or TLR7siRNA administration (n=6). **(C).** Representative images of western blot and statistical analysis of RyR2 expression (n=6). **(D).** qPCR analysis of mRNA level of RyR2 (n=4). Normalized to β-Actin. **(E)** Representative images of western blot and statistical analysis of Serca expression (n=6). **(F)** qPCR analysis of mRNA level for the gene coding for Serca (Atp2a2) (n=4). Normalized to β-Actin. The data are shown as the mean ± SEM, with each data point representing a cell sample. * indicates *p* < 0.05, ** *p* < 0.01, and *** *p* < 0.001.


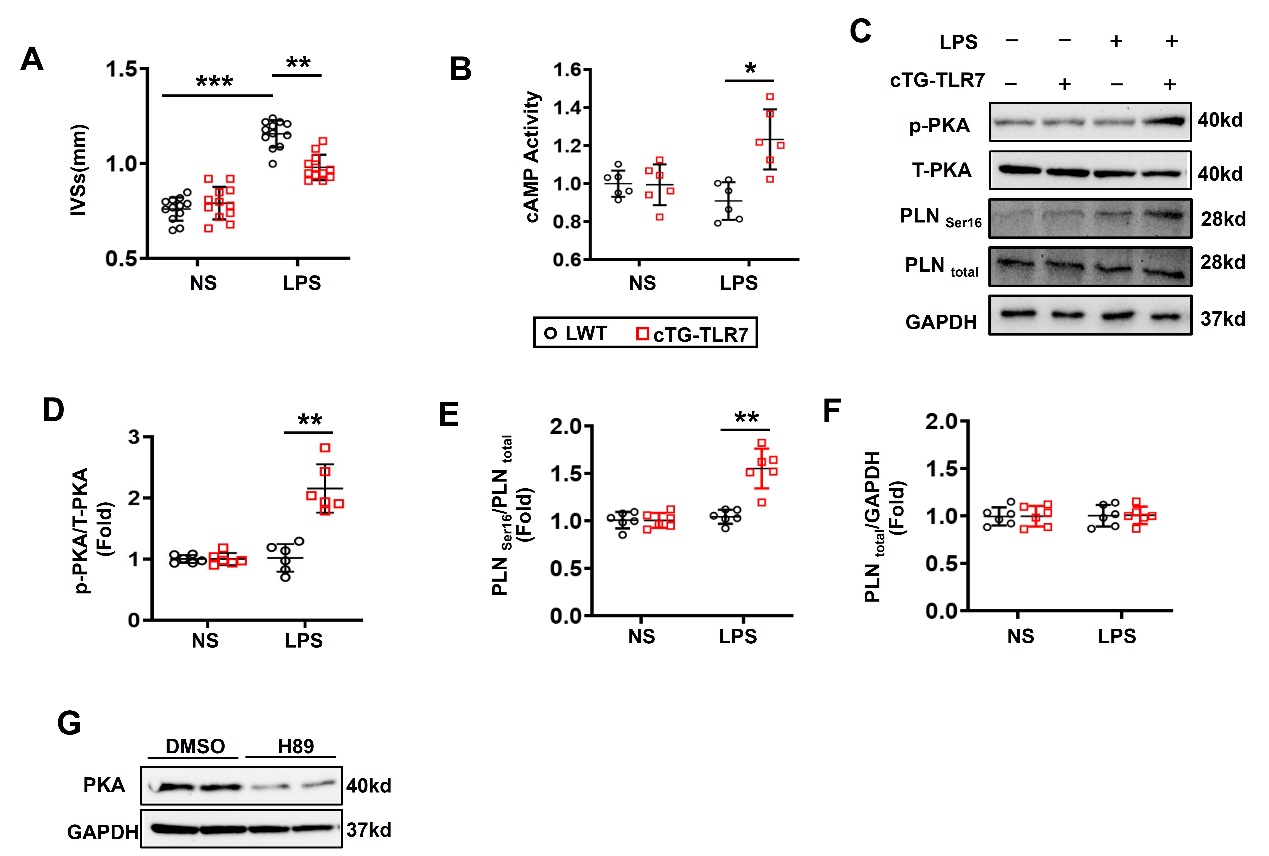


**Supplemental Figure 6:** Echocardiographic examination was used to evaluate cardiac function when LPS administrated for 24 h (n=10-12). **(A)** Interventricular septal systolic thickness (IVSs) was calculated from echocardiography. **(B).** cAMP activity was detected by cAMP-GloTM assay (Promega) and a GLOMAXTM 96 microplate luminometer (Promega) (n=6). **(C-F).** Representative images of western blot and statistical analysis of phosphorylated PKA, total PKA, PLN, phosphorylated Serine 16-PLN expression (n=6). (G). Representative images of western blot of PKA after H89 treatment. The data show as the mean±SEM, with each data point representing a mouse. * indicates *p* < 0.05, ** *p* < 0.01, and *** *p* < 0.001.

**Supplementary Reference**

1. Lee A.S., Ghoreishi M., Cheng W.K., Chang T.Y., Zhang Y.Q., Dutz J.P.,Toll-like receptor 7 stimulation promotes autoimmune diabetes in the NOD mouse. Diabetologia 2011. 54 (6):1407-1416.

2. Zhang Z., Zhang T., Ge Y., Tang M., Ma W., Zhang Q. et al,2D gel electrophoresis reveals dynamics of t-loop formation during the cell cycle and t-loop in maintenance regulated by heterochromatin state. J Biol Chem 2019. 294 (16):6645-6656.

3. Kargacin M.E., Kargacin G.J.,Methods for determining cardiac sarcoplasmic reticulum Ca2+ pump kinetics from fura 2 measurements. Am J Physiol 1994. 267 (4 Pt 1):C1145-1151.

4. Pelled D., Lloyd-Evans E., Riebeling C., Jeyakumar M., Platt F.M., Futerman A.H.,Inhibition of calcium uptake via the sarco/endoplasmic reticulum Ca2+-ATPase in a mouse model of Sandhoff disease and prevention by treatment with N-butyldeoxynojirimycin. J Biol Chem 2003. 278 (32):29496-29501.

5. Wang Y., Bruce A.T., Tu C., Ma K., Zeng L., Zheng P. et al,Protein aggregation of SERCA2 mutants associated with Darier disease elicits ER stress and apoptosis in keratinocytes. J Cell Sci 2011. 124 (Pt 21):3568-3580.

6. Parsons J.T., Churn S.B., Kochan L.D., Delorenzo R.J.,Pilocarpine-induced status epilepticus causes N-methyl-D-aspartate receptor-dependent inhibition of microsomal Mg(2+)/Ca(2+) ATPase-mediated Ca(2+) uptake. J Neurochem 2000. 75 (3):1209-1218.

7. Zhang Q.J., Chen H.Z., Wang L., Liu D.P., Hill J.A., Liu Z.P.,The histone trimethyllysine demethylase JMJD2A promotes cardiac hypertrophy in response to hypertrophic stimuli in mice. J Clin Invest 2011. 121 (6):2447-2456.

8. Liu Z.P., Olson E.N.,Suppression of proliferation and cardiomyocyte hypertrophy by CHAMP, a cardiac-specific RNA helicase. Proc Natl Acad Sci U S A 2002. 99 (4):2043-2048.
